# Optimal Design of Acid Gas-to-Syngas (AG2S) Technology: Process Optimization and Surrogate Modeling

**DOI:** 10.1021/acs.iecr.5c05101

**Published:** 2026-04-07

**Authors:** Simone Caspani, Luis Felipe Sánchez, Mattia Vallerio, Flavio Manenti

**Affiliations:** Department of Chemistry, Materials and Chemical Engineering “Giulio Natta”, 18981 Politecnico di Milano, Piazza Leonardo da Vinci 32, 20133 Milano, Italy

## Abstract

The management of acid gases (i.e., H_2_S and CO_2_) is a fundamental requirement in process plants, such as refineries. Current strategies typically do not exploit the hydrogen content in hydrogen sulfide, which is usually burned. The acid gas-to-syngas (AG2S) technology represents an innovative approach for converting a mixture of hydrogen sulfide (H_2_S) and carbon dioxide (CO_2_) into syngas (H_2_ and CO), with potential applications in fuels and chemical synthesis. This work investigates the optimal process design of AG2S in terms of the H_2_S/CO_2_ and H_2_S/O_2_ feed molar ratios to maximize syngas production. The study combines detailed kinetic and thermodynamic modeling to obtain a comprehensive process simulation, which serves as the basis for generating accurate surrogate models trained on flowsheet simulation data via a design of experiments (DoE) approach. These models allow for a reliable prediction of H_2_S conversion, syngas flow rate, H_2_/CO ratio, and selectivity. The results highlight limitations imposed by relatively low H_2_/CO ratios for downstream applications and illustrate the trade-off between syngas quality and quantity.

## Graphical abstract

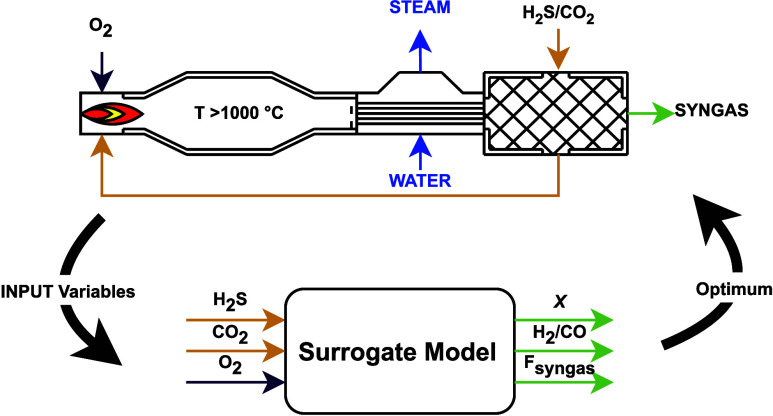

## Introduction

The global energy sector is facing a dual challenge: growing demand for oil and gas and the progressive depletion of conventional, low-sulfur (“sweet”) reserves. As a result, the use of sulfur-rich or “sour” oils has been increasing in the last years.^1^ Moreover, due to increasingly stringent environmental regulations, crude oils undergo deep desulfurization through specific technologies such as hydrotreating and hydrofinishing.^2^ The hydrofinishing process involves catalytic reactions carried out at high pressure and temperature in the presence of hydrogen^3^ and is a necessary step for removing major oil contaminants and improving the final product quality. Consequently, sulfur in the crude oil is converted to H_2_S. These processes are both energy-intensive and highly hydrogen-demanding. As a consequence, refineries are currently managing larger amounts of H_2_S.

Acid gas is primarily a mixture of hydrogen sulfide (H_2_S) and carbon dioxide (CO_2_), often containing small amounts of hydrocarbon gases and, in most cases, water vapor. H_2_S must be treated because it is highly toxic and corrosive to pipelines. CO_2_, on the other hand, is removed since it has no heating value and reduces the overall quality of the natural gas.^4^ Historically, gas producers could flare or incinerate acid gases; however, increasing environmental concerns over sulfur dioxide (SO_2_) emissions have made the flaring of even small quantities of acid gas largely unacceptable. Consequently, the H_2_S recovered from acid gas removal units must be either further processed or reinjected into underground reservoirs. The most commonly adopted processing route is its conversion to elemental sulfur, which is widely implemented in industrial contexts via the Claus process. It involves two main chemical steps, which can be represented by the following reaction kinetics:

1 $$2H_{2}S+3O_{2}\to 2{\mathrm{SO}}_{2}+2H_{2}O,\Delta H_{\mathrm{reaction}}^{0}=-1010.8\;\mathrm{kJ}/\mathrm{mol}$$

2 $$2H_{2}S+{\mathrm{SO}}_{2}\to 3S+2H_{2}O,\Delta H_{\mathrm{reaction}}^{0}=+179.6\;\mathrm{kJ}/\mathrm{mol}$$

In the Claus process, first, about one-third of the hydrogen sulfide in the gas is combusted (eq 1) to generate the sulfur dioxide required for the subsequent reaction (eq 2). The remaining H_2_S reacts with this SO_2_ in the presence of a catalyst, resulting in the formation of elemental sulfur. Through this combination of partial oxidation and catalytic reaction, the Claus process converts the hydrogen sulfide recovered into pure sulfur.^5^ Although it is a mature and commonly used technology for the recovery of sulfur and energy from acid gases, the Claus process has low thermal stage efficiency (i.e., approximately 50–70 mol % of H_2_S converted).^6^

The demand for elemental sulfur is less than the potential supply from the involuntary production of it due to the large sulfur contents in sour gas and crude oils.^1^

Given the low market price of sulfur, the adoption of sulfur recovery units for the conversion of hydrogen sulfide to elemental sulfur is generally not considered economically viable. At last, the hydrogen content of H_2_S remains unexploited, representing a missed opportunity for hydrogen valorization. To this purpose, developing an approach that ensures both environmental sustainability and economic feasibility is essential for the effective management of acid gas.^4^

Considering the information above, acid gas-to-syngas (AG2S) technology is a potential alternative to the conventional route for converting H2S into synthesis gas (syngas). Compared to the traditional Claus process, AG2S technology provides an integrated solution for acid gas utilization and desulfurization,^7^ by combining syngas production and sulfur recovery in a single process. This approach enables the simultaneous treatment of acid gas streams and the generation of a valuable feedstock for fuels and chemicals.

The AG2S technology has been conceptualized and preliminarily investigated by Manenti and Bassani et al.^8–10^ The technical feasibility of the process has already been assessed through process simulation and detailed modeling of the reactive unit, highlighting the potential of this technology. However, a comprehensive investigation of the main process parameters in terms of feed streams is still lacking. Accurate modeling is key to investigating the best operating conditions and improving process efficiency and design. A detailed model requires high computational resources, and sometimes, the exact structure is unknown. To overcome these challenges, surrogate models provide simplified mathematical representations of the main system behavior while significantly reducing computational effort. The development and application of surrogate models, derived from flowsheet simulation data through a design of experiments methodology, allow the systematic optimization of operating conditions, which represents the key aspect of this study. These models enable accurate prediction of H_2_S conversion, syngas flow rate, H_2_/CO ratio, and selectivity, supporting a comprehensive investigation of the influence of process parameters on the process performance, and highlighting the trade-off between syngas quantity and quality.

This work proposes to model the AG2S technology by developing an accurate surrogate model, enabling the systematic mapping and analysis of system behavior across a range of operating conditions. An optimization is performed to identify feed compositions of H_2_S, O_2_, and CO_2_ that maximize both syngas quality and quantity. These outcomes provide insights into the trade-offs between syngas yield and composition, support the definition of operative strategies, and offer a flexible and efficient tool for rapidly representing and exploring all conditions within the domain of interest through an innovative surrogate modeling procedure.

## AG2S Process Layout

AG2S technology (Figure 1) is an innovative process for the production of syngas from feed streams comprising H_2_S, CO_2_, and O_2_. It exploits the hydrogen content of H_2_S as a reducing agent for CO_2_.^10^ The core of the technology is the regenerative thermal reactor (RTR).

**Figure 1:**
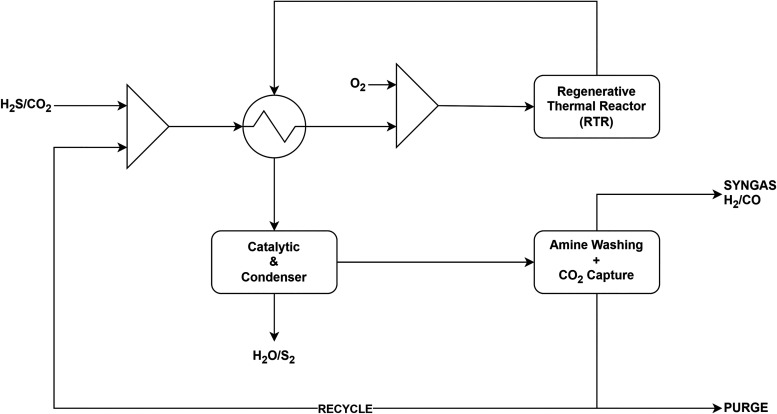
Block flow diagram of the AG2S process.

The inlet streams consist of H_2_S and CO_2_, commonly referred to as “acid gases,” along with oxygen to support combustion, which is needed to ensure the thermal conditions for this endothermic process. The energetic supply is provided by the exothermic oxidation of a portion of H_2_S to SO_2_, according to the following reaction scheme

3 $$2H_{2}S+3O_{2}\to 2{\mathrm{SO}}_{2}+2H_{2}O,\Delta H_{\mathrm{reaction}}^{0}=-1010.8\;\mathrm{kJ}/\mathrm{mol}$$

Temperatures reached in the combustion phase are in the range 1150–1350 °C. Under these conditions, the reaction of H_2_S pyrolysis is considerable to occur, becoming significant above 850 °C and reaching nearly 50% of equilibrium conversion at 1050 °C.^11^ The conversion of H_2_S and CO_2_ into syngas is achieved through an oxy-reduction reaction. The overall theoretical reaction scheme is reported below.

4 $$2H_{2}S+{\mathrm{CO}}_{2}\to H_{2}+\mathrm{CO}+S_{2}+H_{2}O,\Delta H_{\mathrm{reaction}}^{0}=+107.6\;\mathrm{kJ}/\mathrm{mol}$$

Carbon dioxide (CO_2_) is reduced to carbon monoxide (CO) (Reaction: [Disp-formula eq4]), while hydrogen is produced through the pyrolysis of H_2_S (eq 5).^12,13^ As a result, syngas is produced. Byproducts of the process include sulfur, sulfur monoxide, and water. The main process reaction (eq 4) takes place in the RTR, the core component of the plant, as described in the dedicated section.

5 $$H_{2}S\to H_{2}+\frac{1}{2}S_{2},\Delta H_{\mathrm{reaction}}^{0}=+20.4\;\mathrm{kJ}/\mathrm{mol}$$

Downstream from the RTR, the outlet gas stream is then processed through a catalytic section performing the purification of syngas. To this purpose, a multistep process is carried out, involving the hydrolysis of carbon disulfide (CS_2_) and carbonyl sulfide (COS) (eqs 6 and 7),^14^ followed by the conventional Claus reaction for *SO*_2_ conversion eq 8. These compounds must be removed since they are toxic and act as typical catalyst poisons.

6 $$\mathrm{COS}+H_{2}O\to H_{2}S+{\mathrm{CO}}_{2},\Delta H_{\mathrm{reaction}}^{0}=-74.4\;\mathrm{kJ}/\mathrm{mol}$$

7 $${\mathrm{CS}}_{2}+2H_{2}O\to 2H_{2}S+{\mathrm{CO}}_{2},\Delta H_{\mathrm{reaction}}^{0}=-335.1\;\mathrm{kJ}/\mathrm{mol}$$

8 $$2H_{2}S+{\mathrm{SO}}_{2}\to \frac{3}{2}S_{2}+2H_{2}O,\Delta H_{\mathrm{reaction}}^{0}=+121.4\;\mathrm{kJ}/\mathrm{mol}$$

The abatement of COS and CS_2_ is achieved with nearly 100% conversion, and the hydrolysis reactions occur on γ-alumina under mild temperature conditions (i.e., 270 °C).^9^ The removal of water and elemental sulfur is achieved through condensation.^1^

In the last section, further purification of the syngas stream is performed by removing unconverted H_2_S and CO_2_. Acid gases are then recycled back into the process. As highlighted in previous studies,^15^ a well-established example of this purification step can involve chemical solvents or physical ones widely adopted in the process industry and particularly efficient and highly selective toward hydrogen sulfide.

### Regenerative Thermal Reactor

The RTR is primarily composed of a combustion chamber, a waste heat boiler (WHB), and a gas–gas heat exchanger, as illustrated in Figure 2.

**Figure 2:**
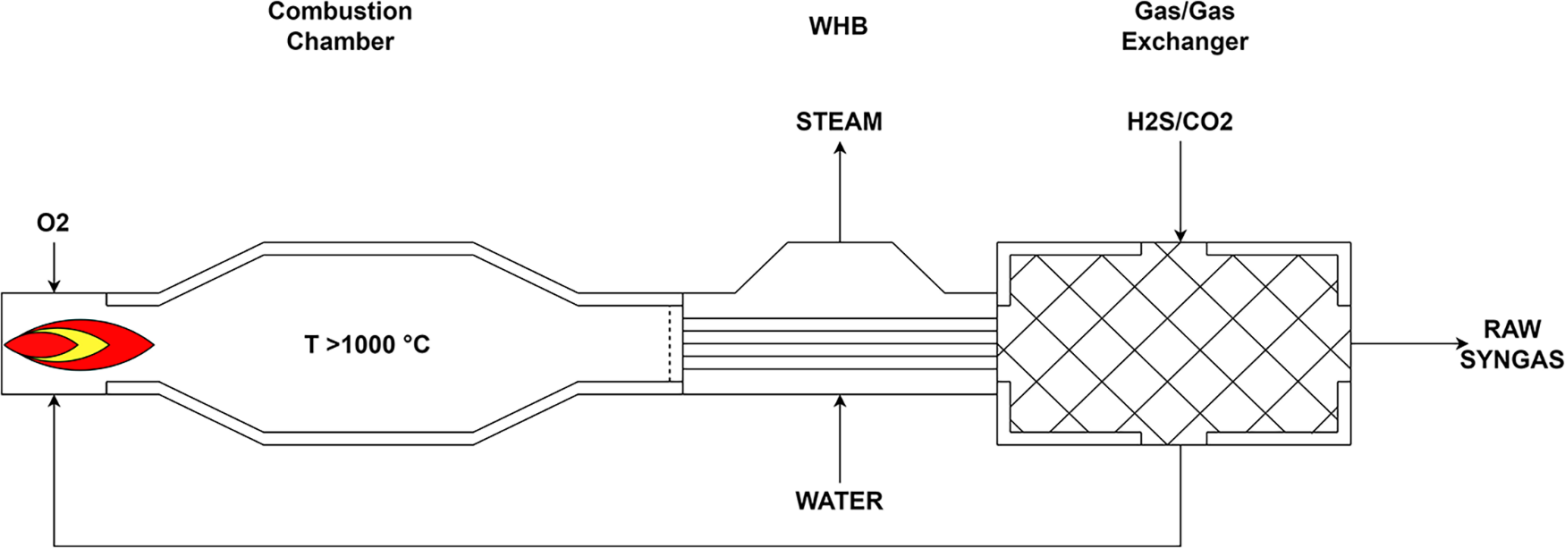
RTR configuration.

The reactor is fed with carbon dioxide and hydrogen sulfide streams at relatively low temperatures (250 °C). These reactants are preheated to approximately 700 °C via heat exchange with the flue gases exiting the furnace (gas/gas exchanger). Once the preheating temperature is achieved, oxygen (at 200 °C) is injected to further raise the temperature to the target range of 1150–1350 °C (combustion chamber). Given the high temperatures and extended residence time (typically >1.0 s), the outlet gas composition is assumed to be close to chemical equilibrium.^11^ As in conventional Claus systems, the heat released during combustion is recovered in the form of high-pressure steam via a Waste Heat Boiler (WHB) to improve overall energy recovery.^16,17^

The cooling of flue gases must occur rapidly to limit undesirable recombination reactions, involving the formation of hydrogen sulfide and carbonyl sulfide (eqs 9 and 10).

9 $$H_{2}+\frac{1}{2}S_{2}\to H_{2}S,\Delta H_{\mathrm{reaction}}^{0}=-20.6\;\mathrm{kJ}/\mathrm{mol}$$

10 $$\mathrm{CO}+\frac{1}{2}S_{2}\to \mathrm{COS},\Delta H_{\mathrm{reaction}}^{0}=-31.6\;\mathrm{kJ}/\mathrm{mol}$$

An effective quenching of the S_2_, H_2_ reassociation reaction would also result in an increase in available H_2_, while preventing COS formation, reducing sulfur loss, and enhancing sulfur recovery.^18^ The quench temperature is approximately 900 °C, with fast quench times (<0.1 s). Recombination reactions (eq 9 and 10) are also exothermic, resulting in an additional heat load. This can influence the thermal duty of the boiler, potentially impacting the design and sizing of the unit.^19^

The flue gases are further cooled to a temperature around 350 °C by transferring heat to the incoming reactants in the gas–gas heat exchanger and then processed to the purification section. This integrated heat recovery strategy reduces the thermal duty of the reactor system and lowers the oxygen demand compared with conventional thermal reactors.

## Methods

### Process Simulation

The AG2S process was simulated in Aspen HYSYS employing the Peng–Robinson–Stryjek–Vera (PRSV) equation of state for the entire process framework, with the exception of the furnace and the waste heat boiler (WHB) of the RTR unit, and implemented as adiabatic plug flow and nonisothermal reactors, respectively, modeled using a dedicated C++-based software developed at Politecnico di Milano for sulfur chemistry modeling. The selected kinetic scheme consists of three distinct subsets of reactions describing the kinetics of carbon and sulfur.^20,21^ This also allows us to predict the formations of minor species such as the organosulfur compounds like COS and CS_2_.^8^ Achieving the quench temperature at the outlet of the WHB (Figure 2) is fundamental as it ensures that recombination reactions are limited and do not affect process performance. Therefore, the gas–gas unit is simulated as a simple process heat exchanger in Aspen HYSYS. The detailed kinetic mechanism^11^ describing RTR reactions is integrated within Aspen HYSYS through MATLAB, enabling the incorporation of detailed kinetics into nonideal reactor models. Moving to the catalytic and condensing section (Figure 1), condensation occurs through a multistage sequence. In the initial stage, the process gas is sufficiently cooled to promote condensation and subsequent removal of water vapor generated during the combustion of hydrogen sulfide. The gas stream, partially depleted of water, then passes through the catalytic Claus reactors, where the reversible reaction between hydrogen sulfide and sulfur dioxide is established, leading to the formation of elemental sulfur and additional water. Each catalytic stage, simulated as an equilibrium reactor, is followed by a dedicated condenser. In addition to the main Claus reaction, an equilibrium exists among the different sulfur allotropes in the gas phase. Furthermore, a conversion reactor is introduced in the simulation to take into account hydrolysis reactions. At the outlet of the catalytic section, complete removal of COS and CS_2_ is achieved. Downstream the catalytic section, a further separation is required for the removal of unconverted acid gases. For simplicity, such a unit is simulated as an ideal separation, where H_2_S and CO_2_ are fully recovered and recycled back to the RTR. Thus, the syngas stream considered is free from contaminants. Following the recycle stream, another component splitter is introduced as a CO_2_ purge. In closed-loop systems, this is commonly used to remove inerts and impurities that accumulate, stabilizing the feed composition.

In the case study considered, a feed flow rate of 2250 kg/h of pure H_2_S is processed, while the relative amounts of CO_2_ and O_2_ are adjusted according to the desired ratios of interest. The selected H_2_S flow corresponds to a global sulfur production of approximately 2 t/h, which is typical for a medium-sized sulfur recovery unit commonly present in the process industry.

### Surrogate Model

Surrogate models are simplified approximations of more complex, higher-order models used to map the input/output correlation between variables. Commonly, these correlations are fully data-driven in black-box approaches. The mathematical models typically used for this purpose rely on simple linear regressions, polynomial regressions, or more complex approaches.^22–24^ When trained on sufficient, high-quality data, they can provide good prediction accuracy across a wide range of applications.^25–27^

The construction of a surrogate model involves four main steps: designing the experiments, running the experiments, training the models, and evaluating the accuracy of the correlation.^28^ The block diagram presented in Figure 3 illustrates the overall workflow employed.

**Figure 3:**
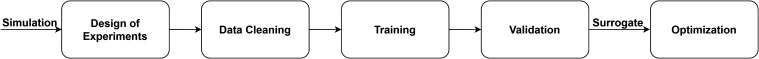
Workflow from process simulation to surrogate-based optimization.

The generation of a surrogate model is based on a data set containing the input–output correlations obtained from experiments or detailed simulations.^29^ In this specific case, the data set was generated from comprehensive process flowsheet simulations.^30^

Once the critical factors, or independent variables, that may influence the system response are identified, an appropriate number of simulations must be carried out. It is desirable to obtain the maximum amount of information with a minimum number of investigated points. The statistical design of experiments (DoE) provides an efficient methodology for planning such investigations.^31,32^ According to a rule of thumb reported in the literature,^33,34^ an adequate number of sampling points can be defined as samples = 10 · #input *variables*.

To avoid the risk of generating meaningless results and to ensure that the predictions remain physically consistent, a data-cleaning step is performed by removing the simulations that do not reach convergence.

At this stage, a mathematical or statistical model is developed to represent the potential dependencies between the considered factors and the response variable.

In the specific case of the AG2S modeling, the Degrees of Freedom (DoF) considered to generate the DoE are presented in Table 1. They include the H_2_S/CO_2_ inlet ratio, which impacts the stoichiometry of reaction 4, and the H_2_S/O_2_ ratio at the inlet of the furnace, affecting the temperature at which the process occurs. In order to ensure compliance with operating limits, proper intervals of investigations are introduced, respectively, for the two variables:

**Table 1:** Degrees of Freedom and Intervals of Investigation

input variable	unit of measure	lower limit	upper limit
*H*_2_ *S*/*CO*_2_	[mol/mol]	1.5	5
*H*_2_ *S*/*O*_2_	[mol/mol]	3.25	5.75

The required number of simulations for surrogate model generation is equal to 20. The sampling technique adopted is the Latin Hypercube,^35^ consisting of a stratified sampling method that generates a near-random set of design variables while ensuring an even distribution across the design space. Sample points are selected to cover the entire design space as uniformly as possible.^36,37^ Specifically to this work, the 20 sample points will fall in the region delimited by the values reported in Table 1.

Once obtained the DoE, the identification of the best overall surrogate model is carried out using cross-validation, with the mean absolute error (MAE) as the evaluation metric.^38^ K-fold cross-validation technique^39^ is adopted to evaluate the surrogate model’s performance. The data set is randomly divided into *K* subsets (or “folds”). One of these subsets is used to validate the model, while the remaining *K* – 1 subsets are used for training. This method provides a more reliable evaluation of model performance and is commonly used with *K* values of 5 or 10.^40^ In this specific work, after completing the process simulations, the data sets are preprocessed to remove potential nonconvergent cases. The resulting data are then split into training (80%) and testing (20%) subsets (i.e., *K* is equal to 5), meaning that the data set is revised every 20/5 = 4 points, 16 points out of 20 are used for the fitting of the model, whereas the remaining 4 are set aside for its validation, ensuring accuracy. The validation data set is isolated and is not used during the cross-validation procedure.^41^ Data management and preprocessing are carried out using Python libraries such as Pandas and NumPy. Several commonly used regression methods in chemical engineering are considered as candidate surrogate models, including linear and polynomial regression, regression trees and random forests, AdaBoost and gradient-boosted regressors, support vector regression, and Kriging with a Radial Basis Function (RBF) kernel. Regression models are implemented using the Scikit-learn Python package, while Kriging is trained using GPyTorch. Model selection is performed via 5-fold cross-validation to ensure robustness and generalization. The performance of each surrogate is evaluated using the mean absolute error (MAE), which provides a consistent and intuitive measure of prediction accuracy. All cross-validation and performance assessments are carried out using Scikit-learn.

### Objective Function and Optimization

The objective function of the study and the consequent optimization rely on different Key Performance Indicators (KPI) focused on syngas production, evaluated in terms of both quantity and quality.

Syngas flow rate *F*_*syngas*_ has been adopted as a key parameter for assessing the productive potential of technology, together with the overall conversion of acid gases and the selectivity of the process toward syngas, as it represents the desired product.

Conversion (*X*) is defined as the fraction of the initial reactant that is transformed during the process. Considering hydrogen sulfide as the reference species, the general expression becomes

11 $$X_{j}=\frac{F_{H_{2}S}^{\mathrm{in},j}-F_{H_{2}S}^{\mathrm{out},j}}{F_{H_{2}S}^{\mathrm{in},j}}$$

where *in* and *out* refer to the generic inlet and outlet streams of the different sections *j* considered—thermal, catalytic, and at the battery limits (overall). However, conversion alone does not indicate which products are formed, and a reaction can exhibit high conversion while generating substantial amounts of undesired byproducts. In this specific analysis, the optimization objective is the overall single-pass conversion, which includes the contributions of the regenerative thermal reactor (RTR), the waste heat boiler (WHB), and the catalytic section (eq 11).

Selectivity (*S*), on the other hand, quantifies the proportion of consumed reactants that are converted specifically to the desired product. It reflects the tendency of the reaction to produce the target compound. On a molar basis, the selectivity of the AG2S process is defined as follows

12 $$S_{\mathrm{overall}}=\frac{F_{H_{2}}^{\mathrm{out}}+F_{\mathrm{CO}}^{\mathrm{out}}}{F_{H_{2}S}^{\mathrm{in}}+F_{CO_{2}}^{\mathrm{in}}}$$

Concerning the quality of the syngas, the selected parameter is the overall ratio between hydrogen and carbon monoxide (H_2_/CO). The H_2_/CO ratio directly reflects the hydrogen production, representing one of the main indicators of high-quality syngas.^42^ A high value is desirable for the synthesis of advanced biofuels such as methane or methanol, requiring stoichiometric ratios of 3 and 2, respectively,^43^ as well as for hydrogen production, which is widely acknowledged as a crucial element in sustainable energy development.^9,44,45^

In addition, a multiobjective optimization was performed to identify an appropriate trade-off between the quality and quantity parameters of the process products, namely, the syngas flow rate and the overall H_2_/CO ratio.

The optimization was conducted using normalized variables, defined as

13 $$Z_{\mathrm{scaled}}=\frac{Z-Z_{\min}}{Z_{\max}-Z_{\min}}$$

where *Z* represents a generic parameter under investigation. This normalization ensures that all variables fall within the same numerical range (0 to 1), thus assigning equal importance to each parameter. Such scaling prevents features with larger magnitudes from disproportionately influencing the optimization results, ensuring a balanced contribution from all objectives.^46,47^ The final optimization criterion is based on the minimization of the sum of the normalized variables.

The optimization of AG2S technology process parameters is subjected to constraints imposed by the operating conditions. Specifically, these include the furnace operating temperature and the outlet temperature from the Waste Heat Boiler.^9^ Maintaining the furnace temperature within the specified range is crucial (eq 14) to ensure a sufficient and consistent energy supply to the process since any deviation from the optimal temperature could significantly affect the reaction kinetics, overall process performances, and yield. An important constraint also lies in the outlet temperature of the WHB, which must guarantee quenching of the recombination reactions, while still ensuring a suitable Δ*T*_*approach*_ that allows an effective heat exchange in the subsequent gas/gas heat exchanger, aimed at preheating the incoming stream to 700 °C. The operating constraints considered in the objective function are as follows:

1200C°<Tout,furnace<1350C° 14

Tout,WHB<900C° 15

16 $$X_{\mathrm{catalytic}}<0$$

The latter constraint is introduced to describe hydrolysis reactions (eqs 6 and 7) taking place in the catalytic section of the process, leading to the formation of hydrogen sulfide.

Optimization studies are conducted using the NOMAD software, which implements the Mesh Adaptive Direct Search (MADS) algorithm, a derivative-free method suitable for solving black-box problems with general nonlinear constraints (Figure 4).^48,49^

**Figure 4:**
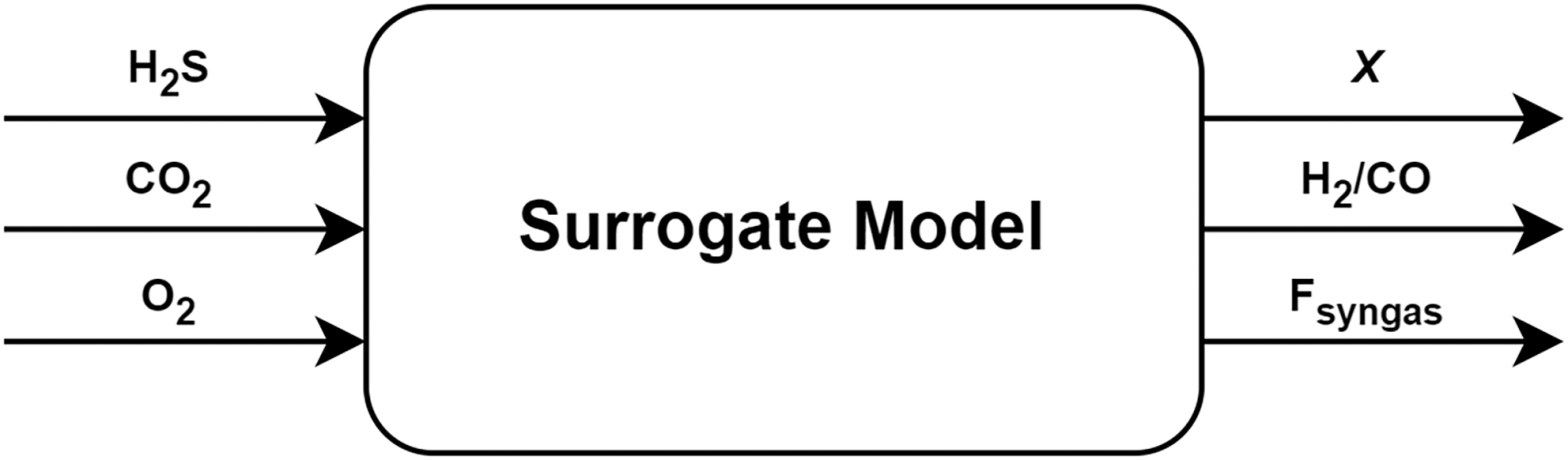
Surrogate model: black-box approach.

All of the response variables considered in the analysis are presented in Table 2. The list includes both performance indicators and operating variables. As depicted, for each variable, there is a model that better describes its behavior.

**Table 2:** Predictive Models

response variable	UoM	predictive model
*T*_*out*, *furnace*_	[°C]	Third Order Polynomial Regression
*T*_*out*, *WHB*_	[°C]	Second Order Polynomial Regression
*X*_*thermal*_	[−]	Second Order Polynomial Regression
*X*_*catalytic*_	[−]	Kriging
*X*_*overall*_	[−]	Third Order Polynomial Regression
*H*_2_/*CO*_*overall*_	[−]	Third Order Polynomial Regression
*F*_*syngas*_	[kg/h]	Third Order Polynomial Regression
*F*_*syngas*_	[kmol/h]	Third Order Polynomial Regression
*S*_*overall*_	[−]	Kriging

According to the current study, the most overall accurate surrogate models, for the main process variables considered, are the second and third-order polynomial regressions, whereas for the overall conversion and selectivity, it has been found that the most suitable model is the Kriging.^50^ The quality of a predictive model is assessed through the evaluation of the mean absolute error (MAE), widely used in regression models.^38^

### Digital Tools

The process framework was simulated in Aspen HYSYS v.11, a commercial process simulation software. The nonconventional unit (i.e., RTR) is modeled using a dedicated C++-based software developed at Politecnico di Milano. This tool is designed to handle sulfur-containing compounds and to describe thermal degradation reactions in detail.^51,52^ The different simulation environments were coupled through a MATLAB connector, enabling data exchange and iterative convergence between the models. The simulation results were finally used.

## Results and Discussion

### DoE and Surrogate Model Accuracy

The Design of Experiments (DoE) strategy described provided 20 combinations of H_2_S/CO_2_ and H_2_S/O_2_ ratios to be investigated. The results of the simulations performed under the conditions defined by the DoE are reported in detail in Table 3. All of the main KPIs under investigation were evaluated and subsequently used to train the surrogate model.

**Table 3:** Simulation Results Employed in the DoE Procedure

# simul.	*H*_2_ *S*/*CO*_2_	*H*_2_ *S*/*O*_2_	*T*_out,furnace_ [°C]	*T*_out,WHB_ [°C]	*X*_thermal_ [%]	*X*_catalytic_ [%]	*X*_overall_ [%]
1	4.86	4.55	1313	835.8	61.28	–4.895	59.38
2	4.51	5.13	1240	784.6	57.45	–6.413	54.72
3	4.29	5.57	1196	754.7	54.33	–6.550	51.34
4	3.36	5.31	1205	753.7	56.51	–7.242	53.36
5	3.22	5.68	1171	731.7	54.74	–7.181	51.49
6	2.74	5.46	1179	733.7	56.52	–7.775	53.14
7	2.93	5.02	1224	768.7	58.81	–7.604	55.68
8	2.41	4.98	1213	756.7	60.25	–8.343	56.93
9	2.07	4.75	1222	758.7	62.71	–8.772	59.44
10	1.54	4.87	1178	723.0	63.73	–10.33	59.98
11	1.69	4.03	1282	791.7	69.37	–4.476	68.00
12	1.93	3.46	1386	867.8	72.14	10.33	75.02
13	2.36	3.54	1403	886.8	70.24	6.680	72.23
14	2.57	3.95	1346	845.8	67.05	–1.619	66.52
15	3.53	4.34	1320	838.8	62.85	–4.646	61.13
16	3.95	4.47	1311	835.8	61.32	–5.029	59.38
17	3.96	4.14	1359	869.8	63.34	–2.603	62.38
18	3.64	3.84	1401	895.8	65.73	0.6013	65.93
19	4.40	3.63	1459	942.8	66.38	3.785	67.66
20	4.77	3.29	1544	998.8	68.87	10.14	72.03

The surrogate models developed from the simulations performed were subsequently evaluated in terms of accuracy. The parity plot shown in Figure 5 illustrates the agreement between the surrogate model predictions and the simulation results. It can be observed that, for each variable, the adopted model is able to reproduce the actual values with an accuracy exceeding 95% (i.e., with an error margin below 5%).^27^

**Figure 5:**
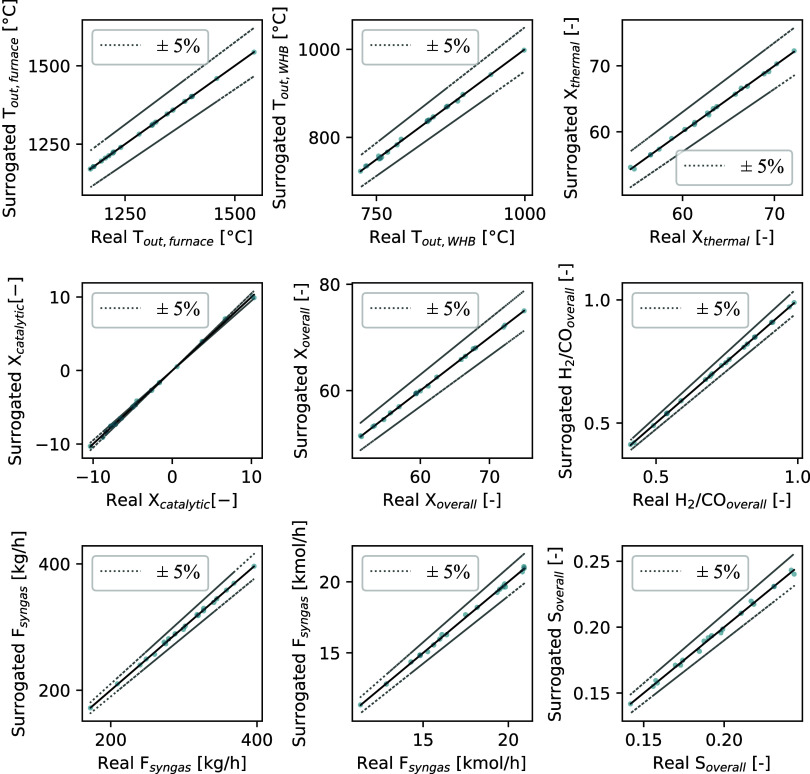
Parity plot comparing surrogate predictions and actual values. Dashed lines indicate ±5% relative error.

### Sensitivity Analysis

Once the best surrogate model was selected, a what-if analysis was carried out on the main process parameters and KPIs to map and highlight the potential impacts of different process variables on the quality and quantity of the produced syngas.^53^ The input parameters are systematically varied to evaluate their influence on the process outcomes. In this study, variations in the design ratios H_2_S/CO_2_ and H_2_S/O_2_ were analyzed within their respective investigation ranges, previously defined (Table 1).

Once the best surrogate model was selected, a what-if analysis was carried out on the main process parameters and KPIs to map and highlight the potential impacts of different process variables on the quality and quantity of the produced syngas.^53^ The input parameters are systematically varied to evaluate their influence on the process outcomes, with the combined effect of both H_2_S/CO_2_ and H_2_S/O_2_ ratios captured across the investigated domain, thereby inherently accounting for their interaction effects.

As reported in Figure 6a, the highest H_2_/CO ratios occur at high H_2_S/CO_2_ values, reflecting a significant presence of hydrogen sulfide that can be converted through reactions 4 and 5. Under these conditions, two regions can be identified where hydrogen production is enhanced: at low and high H_2_S/O_2_ values, respectively. Consequently, the trend of the investigated parameters exhibits a minimum at intermediate H_2_S/O_2_ values. The H_2_S/O_2_ parameter is strictly correlated to the thermal decomposition of H_2_S. Starting from the first optimal scenario, a minimal value of H_2_S/O_2_ corresponds to a high oxygen presence, implying that elevated temperatures are reached due to combustion. Considering the thermodynamics of the reactions involved in hydrogen production, both H_2_S pyrolysis and the oxy-reduction reaction are endothermic and favored at high temperature. While in the scenario where the H_2_S/O_2_ ratio is high, the limited availability of O_2_ constrains H_2_S combustion, leading to a reduced extent of the reaction. Due to the abundant presence of H_2_S, a larger fraction is available for reactions 4 and 5, accounting for the elevated hydrogen production observed under these conditions.

**Figure 6:**
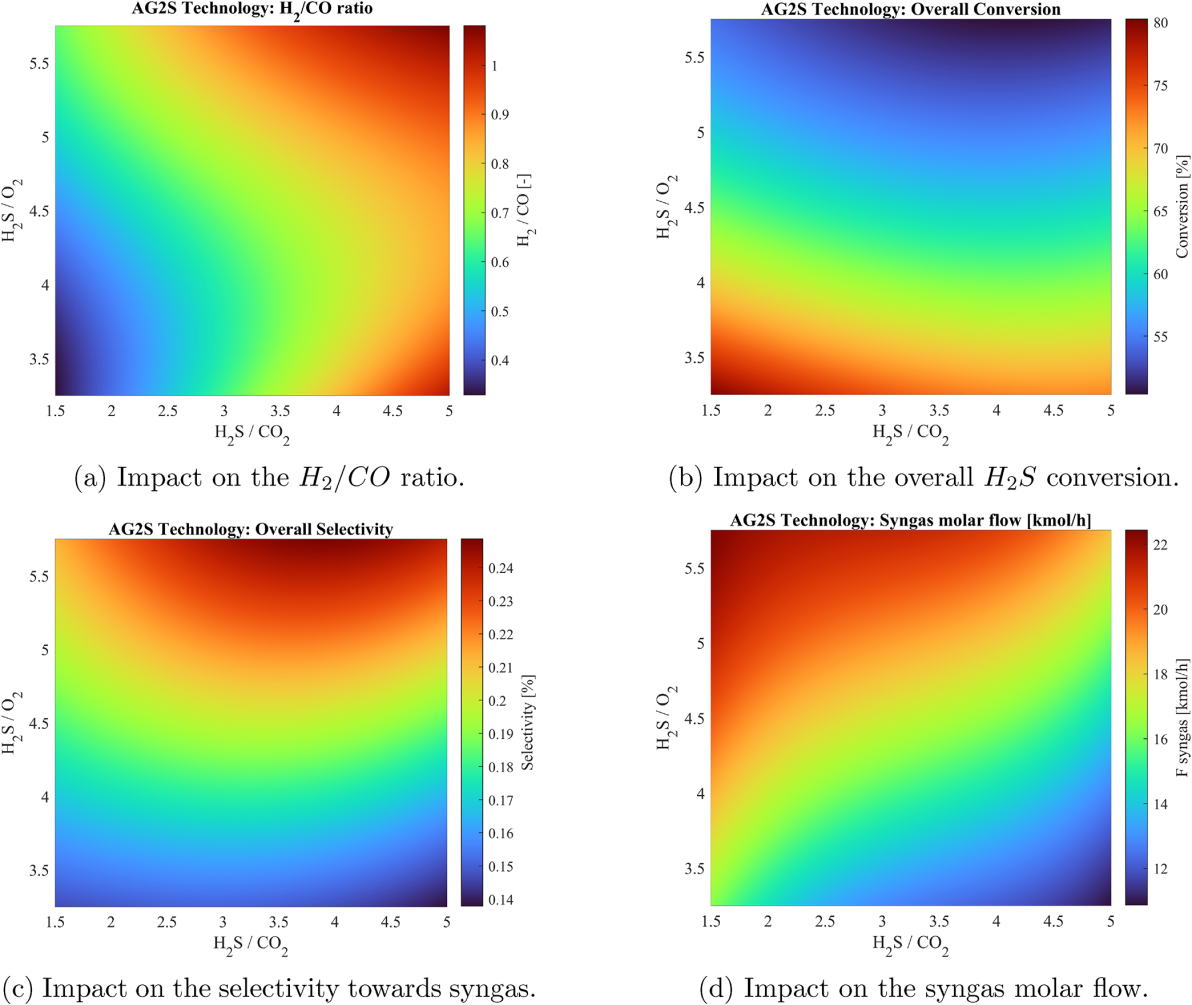
Sensitivity analysis results for the main performance indicators.

The hydrogen sulfide conversion in the RTR unit ranges from around 50% up to 80%, as illustrated in Figure 6b. Higher H_2_S conversion values are obtained at low H_2_S/O_2_ ratios under conditions of oxygen abundance. However, in this situation, the high conversion of H_2_S is mainly due to the predominance of the combustion reaction 3. This implies that syngas production is not the preferred reaction pathway occurring. Higher values of the H_2_S/O_2_ ratio correspond to a decrease in the relative amount of oxygen available, reducing the overall combustion potential of the system. Since temperature is a key driving force for the kinetics of the reactions involved, the reduction of oxygen content directly translates into slower reaction rates and lower conversions of hydrogen sulfide. A decreasing trend in conversion can be observed, as well, by fixing the H_2_S/O_2_ values and increasing the H_2_S/CO_2_ ratios. This behavior is explained by the fact that while the H_2_S content increases, the available amount of oxygen for combustion remains fixed. As a result, the furnace temperature decreases, lowering the overall conversion.

The influence of the design parameters on the molar selectivity toward syngas is depicted in Figure 6c. The trend is opposite to that of conversion (Figure 6b). In a typical AG2S syngas production scenario, a low H_2_S/O_2_ ratio corresponds to an excess of oxygen, resulting in high conversion of H_2_S since nearly all of it reacts. However, under these conditions, the majority of H_2_S undergoes complete combustion to form SO_2_ and H_2_O rather than syngas; therefore, the selectivity toward syngas remains low despite the high overall conversion. On the contrary, at a high H_2_S/O_2_ ratio, oxygen becomes the limiting reactant, leading to a decrease in the overall H_2_S conversion. However, under these conditions, the reaction environment favors partial reactions that produce CO and H_2_, and the selectivity toward syngas increases even though the total H_2_S conversion is lower. At a high H_2_S/CO_2_ ratio, H_2_S is in excess relative to CO_2_, so the reaction environment is more favorable for the desired reaction ([Disp-formula eq4]). Competing pathways are less favored, leading to an increase in the selectivity toward syngas.

Figure 6d reports the *F*_*syngas*_ trends and provides insight into the overall productivity of the process. With respect to the H_2_S/CO_2_ parameter, a relative excess of CO_2_ is correlated with increased syngas production, in accordance with the trends of selectivity (Figure 6c). The abundance of carbon dioxide, as a reactant in reaction 4, makes this kinetic pathway the preferred route, promoting syngas formation. Moving to the analysis on the quantity of oxygen, the increasing trend in F_syngas_ as H_2_S/CO_2_ increases is justifiable because the abundance of oxygen promotes the thermal conversion of hydrogen sulfide, resulting in a reduced amount available for syngas production.

As expected, by comparing the quality of syngas (in terms of H_2_/CO) and the quantity *F*_*synga*s_ an opposite trend is observed. With reference to the H_2_S/CO_2_ ratio, it is evident that a significant amount of syngas is produced when CO_2_ is present in excess. However, this implies that H_2_S becomes the limiting reactant, and pyrolysis reactions, which contribute to hydrogen formation, are strongly limited. This occurs especially under conditions of high H_2_S/O_2_ ratios, where the resulting temperatures are lower. Consequently, the maximum syngas production corresponds to a relatively low syngas quality.

The trends of the furnace temperature as a function of process parameters are provided in the for the sake of clarity.

Figure 7 reports the parallel plot showing the different combinations of independent variables and their effects on the main process indicators. The plot investigates only the feasibility region relevant to the process optimization, delimited by the physical constraints imposed previously (i.e., eqs 14–16). The chart highlights and confirms the trends previously discussed in the what-if analysis. An opposite behavior can be observed between the syngas quality (i.e., the H_2_/CO ratio) and its quantity (i.e., overall conversion). High-quality syngas is obtained when a larger amount of H_2_S is injected into the system compared to the other reactants. Although the overall temperature reached in the RTR furnace is lower, the high H_2_S concentration does not limit the extent of the H_2_S pyrolysis reaction eq 5. Conversely, achieving high overall conversion requires a larger oxygen feed, which increases the process temperature and promotes H_2_S combustion; however, this leads to a reduction in syngas quality.

**Figure 7:**
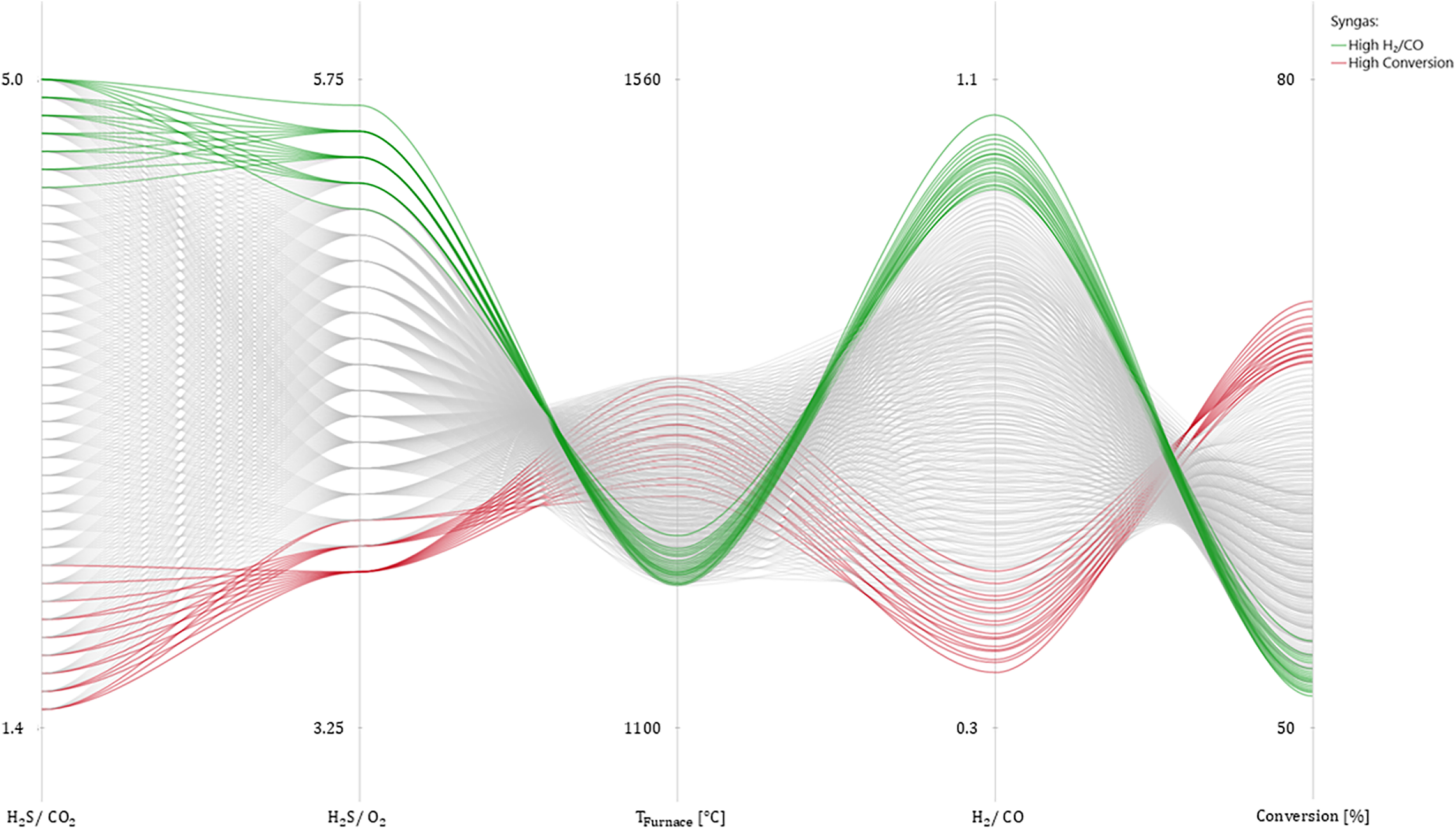
Parallel plot highlighting the dependencies of syngas quality and quantity indicators (H_2_/CO ratio and H_2_S conversion) on the operating variables.

### Optimization

The surrogate model developed was implemented to perform the optimization of the objective functions previously described while ensuring compliance with the physical constraints of the process (i.e., eqs 14–16).

In this work, the optimization focuses on the overall conversion aimed at maximizing H_2_S abatement along with simultaneous hydrogen production. Additional target variables include the H_2_/CO ratio and the syngas flow rate *F*_*syngas*_, which represent the quality of the target product and the production potential of the AG2S technology. The syngas flow rate is also used to evaluate the maximum selectivity of the process toward syngas. The results of the optimization are summarized in Table 4.

**Table 4:** Process Optimization Results

H_2_S/CO_2_	H_2_S/O_2_	*X*_overall_ [%]	H_2_/CO	*F*_syngas_ [kmol/h]	selectivity [%]
1.5	3.82	71.44	0.36	18.17	16
5.0	5.68	51.34	1.06	18.20	24
1.5	4.62	62.23	0.49	20.48	19

As shown in Table 4, high-quality syngas is obtained under conditions where both design parameters assume high values according to the sensitivity analysis performed (Figure 6a). When the H_2_S/O_2_ ratio is high, the limited availability of oxygen restricts the combustion of hydrogen sulfide, thereby reducing the overall reaction extent. The excess H_2_S, however, is available for reactions 4 and 5, which account for the higher hydrogen yield observed under these conditions. In the optimal scenarios, the corresponding H_2_/CO ratio is slightly above 1. Although this value is insufficient for downstream syntheses of chemicals such as methanol or methane, requiring stoichiometric ratios of 2 and 3, respectively. However, if H_2_ is the product of interest, the implementation of the water–gas shift (WGS) reaction could further convert carbon monoxide, enhancing the overall hydrogen yield.

Concerning the single-pass molar conversion of H_2_S, the outcomes are consistent with the conclusions previously derived from the sensitivity analysis (Figure 6b). Optimal performance in the overall conversion is achieved at limited H_2_S/CO_2_ ratios, ensuring that oxygen becomes the excess reactant, thus enhancing conversion due to the higher furnace temperatures. For the same reason, the optimal H_2_S/O_2_ ratio corresponds to a situation in which oxygen is abundant.

Concerning the maximization of the syngas flow rate, it can be noted that the conditions granting optimal productivity show similarities with the point of minimum quality in terms of the H_2_/CO ratio, as reported in Figure 6a. Indeed, the syngas obtained in this scenario is richer in carbon monoxide relative to hydrogen.

According to the sensitivity analysis, giving priority to high-quality syngas means lowering the productivity and vice versa. To this purpose, the balance between the two is investigated through a multiobjective optimization. The optimal conditions leading to a balanced trade-off between the quality and quantity of the syngas produced are, as expected, found at an intermediate situation (Table 5). Although the syngas flow rate value does not markedly deviate from the condition of optimal productivity, the H_2_/CO ratio remains considerably low, particularly.

**Table 5:** Multiobjective Optimization

H_2_S/CO_2_	H_2_S/O_2_	*X*_overall_ [%]	H_2_/CO	*F*_syngas_ [kmol/h]	selectivity [%]
2.4	3.87	67.85	0.51	15.99	16

While both quality and quantity are fundamental parameters within a production process and have therefore been assigned equal weight, in practical applications, the selection of operating conditions is primarily driven by the relative priority attributed to one of them, thus granting greater relevance to the parameter considered most critical.

## Conclusions and Future Developments

This work provides a comprehensive evaluation of the AG2S process as an innovative approach for the valorization of acid gases as reagents in syngas production, highlighting the relevance and versatility of the process in advancing sustainable chemical technologies.

From the detailed modeling activity, carried out through conventional and customized digital tools, it was possible to obtain the input data required to train and validate an accurate surrogate model, enabling sensitivity analysis and process optimization. The analysis allowed us to identify the optimal operating conditions in terms of H_2_S/CO_2_ and H_2_S/O_2_ ratios, considering both the quality and the quantity of the produced syngas. The study highlights that high-quality syngas (i.e., hydrogen rich) is favored at low CO_2_ levels, while the overall conversion and syngas yield are enhanced when a sufficiently high amount of oxygen is supplied to the system.

This work represents a preliminary investigation aimed at exploring and identifying the optimal operating conditions for the AG2S process. The study focuses on analyzing the influence of various process parameters on the performance, providing a foundation for further research and optimization efforts for this process. The analysis highlights preliminary results that can serve as a starting point for the potential scale-up of the technology, whether at the pilot or industrial scale.

Future perspectives for the optimization of this process may include a more detailed analysis of the operating conditions that maximize syngas selectivity, as well as a comprehensive optimization considering both syngas quality and quantity, with appropriate weighting of the variables to achieve the desired quality targets for the product.

Considering the novel nature of the process, giving priority to syngas quality is a critical aspect of AG2S process development before large-scale production can be pursued.^8^ For instance, potential applications of this technology include the development of a low-impact methanol production process,^10^ as well as alternative routes to conventional green hydrogen production. Within this framework, AG2S technology could be integrated into an existing industrial-scale methanol production facility, mitigating reliance on fossil resources and promoting more sustainable production practices, although additional process steps would be needed to adjust the H_2_/CO ratio for chemical synthesis.

## Supplementary Material





## Notes

Kinetic mechanism: Lists the kinetic mechanism used to model the reactions occurring in the RTR unit. Surrogate predictive models: Parameters and coefficients for the models derived during surrogate generation. Furnace temperature trend: Reports the temperature distribution in the RTR furnace as a function of operating conditions. Stream details for maximum conversion conditions: Provides the stream information for a specific case study (PDF)
Surrogato Work flow (ZIP)
