# Supplementary material for: Optimal Design of Acid Gas-to-Syngas (AG2S) Technology: Process Optimization and Surrogate Modeling
Source: Ind Eng Chem Res. 2026 Apr 7;65(15):8006–16. doi: 10.1021/acs.iecr.5c05101 (PMC13127113; doi:10.1021/acs.iecr.5c05101)
Supplement: Supplementary file 1 [file ie5c05101_si_001.pdf]

Supporting Information: 'Optimal Design of Acid Gas-to-Syngas (AG2S™) Technology: Process Optimization and Surrogate Modeling'

Simone Caspani, Luis Felipe Sánchez, Mattia Vallerio, and Flavio Manenti*

Department of Chemistry, Materials and Chemical Engineering "Giulio Natta", Politecnico di Milano, Piazza Leonardo da Vinci 32, 20133 Milano, Italy

E-mail: flavio.manenti@polimi.it

Furnace Temperature Trend

The furnace temperature in the RTR unit ranges from approximately 1100 °C up to 1550 °C, as shown in Figure S1.

A clear trend can be observed with respect to the H_2S/O_2 ratio. Lower H_2S/O_2 values, corresponding to oxygen-rich conditions, lead to higher furnace temperatures due to the enhanced combustion of hydrogen sulfide. Under these conditions, combustion reactions are relevant, and syngas formation is not the preferential reaction pathway. As the H_2S/O_2 ratio increases, the relative availability of oxygen decreases, resulting in a reduced combustion potential and, consequently, lower furnace temperatures. The decrease in oxygen concentration leads to slower reaction rates and lower overall conversion of hydrogen sulfide.

On the other hand, the presence of CO_2 negatively affects the temperature profile through the RTR unit. At fixed H_2S/O_2 conditions, increasing the H_2S/CO_2 ratio corresponds to a lower relative amount of CO_2, which reduces the heat demand associated with endothermic

reactions involving CO_2. As a result, the furnace temperature increases with increasing H_2S/CO_2 ratio.

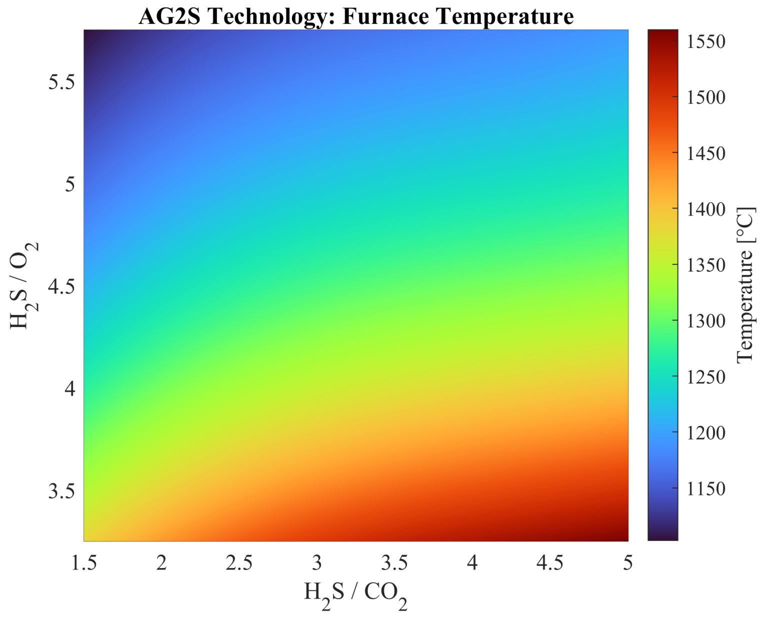

Figure S1: Impact on the furnace temperature.

Surrogate Predictive Models

The surrogate models operate on normalized variables in order to avoid issues related to differences in scale. The input variables are normalized according to Eq. 1. Similarly, the surrogate models return normalized output variables, which must be denormalized to obtain physically meaningful values. Table S1 reports the minimum and maximum values of the ranges for each input and output variable used to perform the normalization and denormalization procedures.

$$Z_{scaled} = \frac{Z - Z_{min}}{Z_{max} - Z_{min}} \tag{1}$$

For each variable, the most accurate surrogate model was identified. For the output variables of interest, the resulting surrogate models consist of second-order and third-order

Table S1: Minimum and maximum values of input and output variables used for normalization and denormalization.

Variable	UoM	Min	Max
H_2S/CO_2	[-]	1.54	4.86
H_2S/CO_2	[-]	3.29	5.68
T_{out} Furnace	[°C]	1171	1544
T_{out} WHB	[°C]	723	998.8
X_{thermal}	[% mol]	54.33	72.14
$X_{\text{catalytic}}$	[% mol]	10.33	-10.33
X_{overall}	[% mol]	51.34	75.02
H_2/CO	[-]	0.4101	0.9874
F_{syngas}	[kg/h]	172.1912	396.0925
F_{syngas}	[kmol/h]	11.322	20.9284
S_{overall}	[-]	0.1418	0.2435

polynomial functions as well as Kriging models, as reported in Table S2.

Table S2: Predictive models.

**Response Variable**	**UoM**	**Predictive Model**
$T_{out,furnace}$	[°C]	Third Order Polynomial Regression
$T_{out,WHB}$	[°C]	Second Order Polynomial Regression
$X_{thermal}$	[-]	Second Order Polynomial Regression
$X_{catalytic}$	[-]	Kriging
$X_{overall}$	[-]	Third Order Polynomial Regression
$H_2/CO_{overall}$	[-]	Third Order Polynomial Regression
F_{syngas}	[kg/h]	Third Order Polynomial Regression
F_{syngas}	[kmol/h]	Third Order Polynomial Regression
$S_{overall}$	[-]	Kriging

Third Order Polynomial Regression

A third-order polynomial regression model includes linear, quadratic, cubic, and interaction terms, allowing the representation of nonlinear behavior within the studied domain. Third-order polynomials are expressed as reported in Eq. 2. The detailed values of the model parameters for each variable are reported in Table S3.

$$P(x, y) = a_0 + a_1x + a_2y + a_3x^2 + a_4xy + a_5y^2 + a_6x^3 + a_7x^2y + a_8xy^2 + a_9y^3 \quad (2)$$

Where x is the normalized H_2S/CO_2 ratio and y is the normalized H_2S/O_2 ratio.

Table S3: Coefficients of the third-order polynomial surrogate models.

Variable	a_0	a_1	a_2	a_3	a_4	a_5	a_6	a_7	a_8	a_9
T_{out} Furnace	0.00000	0.89181	-1.11497	-0.70016	-0.57126	0.54741	0.25143	0.14376	0.21574	0.56290
$X_{overall}$	0.00000	-0.64956	-1.70851	0.27990	0.59483	0.77658	0.05658	-0.09973	-0.32863	1.18441
H_2/CO	0.00000	1.12747	0.03306	-0.02970	-1.11757	1.19276	0.03191	-0.37442	1.05643	-0.12687
F_{syngas}	0.00000	-1.77317	0.71896	1.67642	0.76960	-0.67859	-0.70328	-0.65306	0.16895	0.78484
F_{syngas} (kmol)	0.00000	-1.33524	0.85762	1.66998	0.34013	-0.00799	-0.86102	-0.62817	0.43434	0.51354

Second Order Polynomial Regression

A second-order polynomial regression model includes linear, quadratic and interaction terms, allowing the representation of nonlinear behavior within the studied domain. Second-order polynomials are expressed as reported in Eq. 3. The detailed values of the model parameters for each variable are reported in Table S4.

$$P(x, y) = b_0 + b_1 x + b_2 y + b_3 x^2 + b_4 xy + b_5 y^2 \tag{3}$$

where x is the normalized H_2S/CO_2 ratio and y is the normalized H_2S/O_2 ratio.

Table S4: Coefficients of the second-order polynomial surrogate models.

Variable	b_0	b_1	b_2	b_3	b_4	b_5
T_{out} WHB	0	0.77076	-1.03642	-0.28527	-0.27865	0.51719
$X_{thermal}$	0	-0.78303	-0.96991	0.43654	0.13135	1.16055

Kriging Model

A surrogate model based on Kriging was employed. The model uses a radial basis function (RBF) as the covariance function, which quantifies the similarity between pairs of points based on their distance in the input domain. To account for potential measurement errors or intrinsic variability in the data, a noise term was included in the covariance function. This improves the robustness of the predictions, providing a more reliable surrogate for subsequent analysis or optimization. Eq. 4 shows the detailed expression of Kriging's correlation.

$$k_{\text{noisy}}\big((x_i, y_i), (x_j, y_j)\big) = \sigma_f^2 \exp\left[-\frac{(x_i - x_j)^2}{2\ell^2} - \frac{(y_i - y_j)^2}{2\ell^2}\right] + \sigma_n^2\,\delta_{ij} \tag{4}$$

Where:

- x_i, y_i coordinates of training point i, corresponding to normalized H_2S/CO_2 and H_2S/O_2 ratios.
- x_j, y_j coordinates of training point j, corresponding to normalized H_2S/CO_2 and H_2S/O_2 ratios.
- σ_f^2 signal variance: defines the amplitude of the predicted function variations.
- ℓ length scales; control how fast the function varies in each direction.
- σ_n^2 noise variance: accounts for independent fluctuations in observed data.
- δ_{ij} Kronecker delta: equals 1 if $i = j$, 0 otherwise; applies the noise only on the covariance matrix diagonal.

The details of the parameters obtained for the variables of interest are reported in Table S5, including the estimated average values of the signal variance σ_f^2 and the noise variance σ_n^2.

Table S5: Kriging hyperparameters.

Variable	σ_f^2	ℓ	σ_n^2
$X_{catalytic}$	0.00019	0.67888	0.35009
$S_{overall}$	0.00036	1.36776	0.51082

Stream Detail for Maximum Conversion Conditions

The detailed stream conditions and molar compositions are reported in Table S6. Representative results corresponding to the optimal conversion conditions achieved in the AG2S™ process are presented, referring to Case 1 in Table 4.

Minor discrepancies between the results predicted by the surrogate model and those obtained from the detailed process simulations are observed. These differences fall within the expected accuracy of the surrogate model, with deviations of approximately 5%.

Table S6: Stream detail for maximum conversion conditions.

Stream	UoM	H_2S Feed	CO_2 Feed	O_2 Feed	Out Furnace	Out WHB	Water/Sulphur	Syngas	Recycle	CO_2 Purge
T	[°C]	45	50	200	1258	704	50	50	50	50
P	[kPa]	182.5	182.5	182.5	182.5	182.5	182.5	182.5	182.5	182.5
Flow	[kg h^{-1}]	2250	1937	775	6436	6465	3096	535.8	1498	1336
Flow	[kmol h^{-1}]	66.03	44.02	24.21	186	182.7	–	25.95	39.36	30.35
**Molar composition [mol mol^{-1}]**										
O_2		0.00	0.00	1.00	0.00	0.00	0.00	0.00	0.00	0.00
H_2S		1.00	0.00	0.00	0.10	0.13	0.00	–	0.60	0.00
CO_2		0.00	1.00	0.00	0.22	0.23	0.00	0.00	0.40	1.00
H_2		0.00	0.00	0.00	0.05	0.03	0.00	0.19	0.00	0.00
CO		0.00	0.00	0.00	0.10	0.08	0.00	0.56	0.00	0.00
H_2O		0.00	0.00	0.00	0.33	0.34	0.87	0.25	0.00	0.00
S		0.00	0.00	0.00	0.18	0.16	0.13	0.00	0.00	0.00
COS		0.00	0.00	0.00	0.01	0.02	0.00	0.00	0.00	0.00
SO_2		0.00	0.00	0.00	0.01	0.01	0.00	0.00	0.00	0.00

Kinetic Mechanism

The kinetic model used to simulate the reactions is reported in Tables S7, S8, and S9. The reaction rates are expressed using a modified Arrhenius form:

$$k = A\,T^n \exp\left(-\frac{E}{RT}\right) \tag{5}$$

Table S7: Reaction set — H_2S Pyrolysis

No.	Reaction	A	n	E [cal/mol]
1	$H_2S + M = SH + H + M$	1.76×10^{15}	0.0	64000.0
2	$H_2S + M = H_2 + S + M$	2.00×10^{14}	0.0	66000.0
3	$H_2S + H = SH + H_2$	1.08×10^{11}	0.0	2969.7
4	$H_2S + S = SH + SH$	8.30×10^{13}	0.0	2052.7
5	$H_2S + S = H_2 + S_2$	6.02×10^{12}	0.0	4968.0
6	$H_2S + S = HS_2 + H$	2.00×10^{13}	0.0	7400.1
7	$H + H + M = H_2 + M$	1.87×10^{18}	−1.0	0.0
8	$H + H + H = H_2 + H$	3.20×10^{15}	0.0	0.0
9	$H_2 + M = H + H + M$	2.29×10^{20}	−1.4	104380.0
10	$S + H + M = SH + M$	6.20×10^{16}	−0.6	0.0
11	$S + H_2 = SH + H$	2.00×10^{14}	0.0	76600.0
12	$S + S + M = S_2 + M$	1.20×10^{17}	−1.0	0.0
13	$S_2 + M = 2\,S + M$	4.80×10^{13}	0.0	77103.9
14	$S_2 + H + M = HSS + M$	1.15×10^{25}	−2.8	1665.0
15	$SH + H = H_2 + S$	5.16×10^{14}	0.0	21000.0
16	$SH + S = H + S_2$	1.50×10^{13}	0.0	0.0
17	$SH + SH = S_2 + H_2$	1.28×10^{14}	0.0	0.0
18	$SH + SH = H_2S + S$	1.00×10^{14}	0.0	430.0
19	$HSSH + M = SH + SH + M$	1.40×10^{15}	1.0	57030.0
20	$HSS + H = SH + SH$	1.10×10^{13}	0.4	210.0
21	$HSS + H = H_2 + S_2$	4.19×10^{8}	1.6	472.0
22	$HSS + H = H_2S + S$	1.50×10^{8}	1.6	2149.6
23	$HS_2 + H + M = H_2S_2 + M$	1.00×10^{16}	0.0	0.0
24	$H_2S_2 + H = HS_2 + H_2$	1.20×10^{7}	2.1	715.4
25	$H_2S_2 + S = HS_2 + SH$	2.00×10^{13}	0.0	7400.0

Table S8: Reaction set — H_2S Oxidation

No.	Reaction	A	n	E [cal/mol]
26	$H_2S + O_2 = HSO + OH$	1.00×10^{11}	0.0	49100.0
27	$H_2S + OH = SH + H_2O$	8.70×10^{13}	−0.7	0.0
28	$H_2S + HSO = SH + HSOH$	1.00×10^{13}	0.0	17300.0
29	$H_2S + HOS = SH + HSOH$	1.00×10^{13}	0.0	12500.0
30	$H_2S + SO = HSO + SH$	5.38×10^{3}	3.2	26824.0
31	$SH + OH = HOS + H$	1.00×10^{13}	0.0	7400.0
32	$SH + HSO = S + HSOH$	1.00×10^{11}	0.0	11000.0
33	$H_2 + S_2O = SH + HOS$	1.00×10^{13}	0.0	46000.0
34	$SH + SO = HSO + S$	1.00×10^{13}	0.0	25000.0
35	$HSSO_2 + M = SH + SO_2 + M$	1.00×10^{17}	0.0	3000.0
36	$HSO + S_2 = HSS + SO$	1.00×10^{12}	0.0	3000.0
37	$HSO + H = SH + OH$	4.90×10^{19}	−1.9	1560.0
38	$HSO + OH = H_2O + SO$	1.70×10^{9}	1.0	470.0
39	$HOS + O = H + SO_2$	1.00×10^{14}	0.0	0.0
40	$HSSO + O = SH + SO_2$	1.00×10^{13}	0.0	0.0
41	$HSSO + H = S_2O + H_2$	1.00×10^{13}	0.0	0.0
42	$HSSO + OH = S_2O + H_2O$	1.00×10^{13}	0.0	0.0
43	$HSSO + S = HSS + SO$	1.00×10^{13}	0.0	0.0
44	$HSSO + HSS = S_2O + HSSH$	1.00×10^{13}	0.0	0.0
45	$HSSO + HO_2 = S_2O + H_2O_2$	1.00×10^{13}	0.0	0.0
46	$S_2O + HSO_2 = HSSO + SO_2$	1.00×10^{13}	0.0	32000.0
47	$S_2O + H + M = HSSO + M$	6.42×10^{22}	−2.6	286.6
48	$SO + H + M = HSO + M$	5.00×10^{15}	0.0	0.0
49	$SO^* + M = SO + M$	1.00×10^{13}	0.0	0.0
50	$SO^* + O_2 = SO_2 + O$	1.00×10^{13}	0.0	0.0
51	$SO + O + M = SO_2 + M$	3.20×10^{13}	0.0	0.0
52	$SO + HO_2 = SO_2 + OH$	3.70×10^{3}	2.4	7660.0
53	$SO + SO + M = OSSO + M$	3.23×10^{32}	−5.8	3044.2
54	$S + SO_2 = 2\,SO$	5.88×10^{12}	0.0	9034.0
55	$HSO_2 + O = SO_2 + OH$	1.00×10^{13}	0.0	0.0
56	$HSO_2 + OH = SO_2 + H_2O$	1.00×10^{13}	0.0	0.0
57	$HSO_2 + SH = SO_2 + H_2S$	1.00×10^{13}	0.0	0.0
58	$HSO_2 + S = SO_2 + SH$	1.00×10^{13}	0.0	0.0
59	$SO + SH = S_2 + OH$	1.00×10^{12}	0.0	4320.0
60	$S_2O + O = 2\,SO$	9.27×10^{11}	0.0	0.0
61	$S_2O + S = SO + S_2$	1.00×10^{13}	0.0	0.0
62	$S_2O + SH = HSO + S_2$	1.00×10^{12}	0.0	5000.0
63	$HSS + O = SH + SO$	1.00×10^{14}	0.0	0.0
64	$HSSH + O = HSO + SH$	1.00×10^{14}	0.0	0.0
65	$HSSH + OH = HSS + H_2O$	1.00×10^{14}	0.0	0.0
66	$HSOH + H = HSO + H_2$	1.00×10^{14}	0.0	0.0

Table S9: Reaction set — CO_2, COS, CS_2 formation

No.	Reaction	A	n	E [cal/mol]
67	$CO_2 + M = CO + O + M$	6.06×10^{13}	0.0	104445.5
68	$CO_2 + O = CO + O_2$	1.24×10^{10}	0.0	43830.0
69	$COS + O = CO + SO$	2.00×10^{13}	0.0	7385.0
70	$S + COS = S_2 + CO$	2.95×10^{8}	0.0	3404.0
71	$O + COS = CO_2 + S$	5.00×10^{8}	0.0	10990.1
72	$HCO + M = H + CO + M$	5.70×10^{11}	0.7	14870.0
73	$HCO + H = CO + H_2$	7.34×10^{13}	0.0	0.0
74	$HCO + O = CO + OH$	3.02×10^{13}	0.0	0.0
75	$HCO + O = CO_2 + H$	3.00×10^{13}	0.0	0.0
76	$HCO + OH = CO + H_2O$	1.02×10^{14}	0.0	0.0
77	$HCO + HCO = CH_2O + CO$	1.80×10^{13}	0.0	0.0
78	$HCO + H_2O = CO + H + H_2O$	2.24×10^{18}	−1.0	17000.0
79	$CH_2O + M = HCO + H + M$	3.31×10^{16}	0.0	81000.0
80	$CH_2O + OH = HCO + H_2O$	7.59×10^{12}	0.0	170.0
81	$OH + CO = H + CO_2$	6.76×10^{7}	1.1	70.0
82	$CO + O = CO_2$	5.90×10^{15}	0.0	4100.0
83	$H + H + CO_2 = H_2 + CO_2$	5.50×10^{20}	−2.0	0.0
84	$C + SO_2 = CO + SO$	4.16×10^{13}	0.0	0.0
85	$C + H_2S = CH + SH$	1.20×10^{14}	0.0	8843.7
86	$O + CS = CO + S$	1.63×10^{14}	0.0	1510.3
87	$COS + M = CO + S + M$	1.43×10^{15}	0.0	61007.1
88	$CH + SO = CO + SH$	1.00×10^{13}	0.0	0.0
89	$SO_2 + CO = SO + CO_2$	2.70×10^{12}	0.0	48289.0
90	$H + HCS = CS + H_2$	1.21×10^{14}	0.0	0.0
91	$SH + CS = H + CS_2$	3.23×10^{10}	1.5	495.0
