# Supplementary figures and images for: Optimal Design of Acid Gas-to-Syngas (AG2S) Technology: Process Optimization and Surrogate Modeling
Source: Ind Eng Chem Res. 2026 Apr 7;65(15):8006–16. doi: 10.1021/acs.iecr.5c05101 (PMC13127113; doi:10.1021/acs.iecr.5c05101)

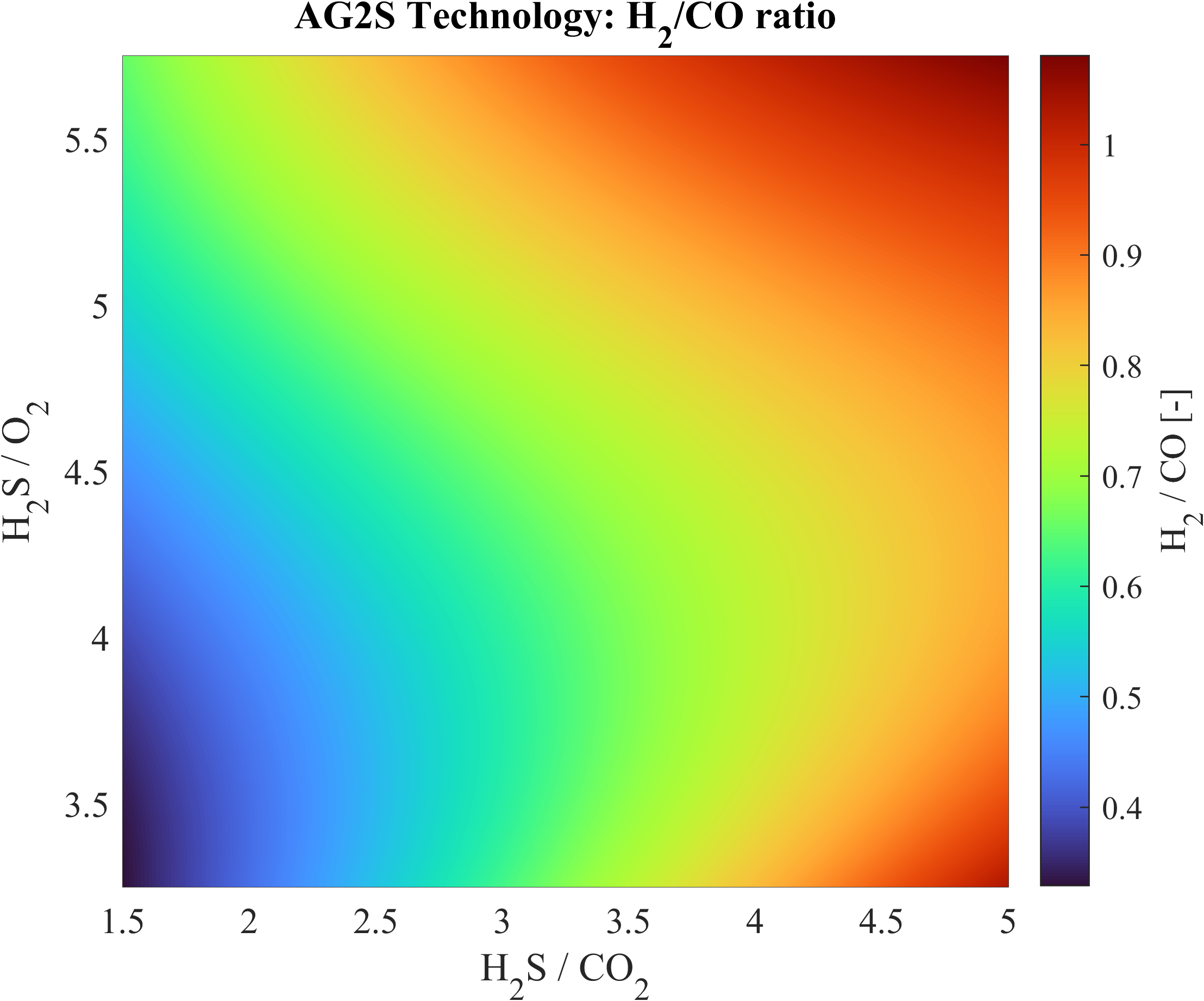

Supplement: Supplementary file 2 [file ie5c05101_si_002.zip › Images/plot_H2toCO.png]

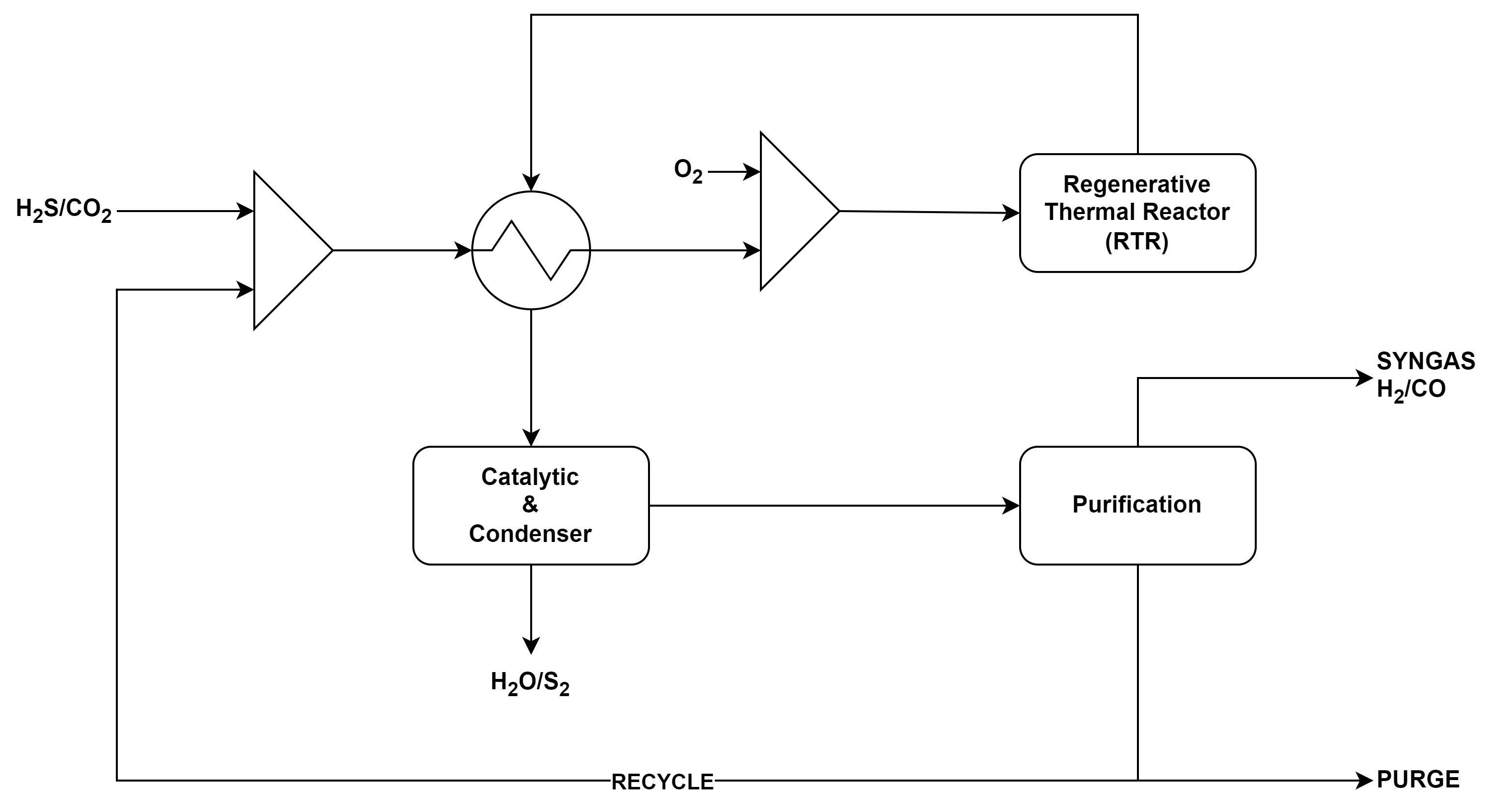

Supplement: Supplementary file 2 [file ie5c05101_si_002.zip › Images/1_OverallScheme.png]

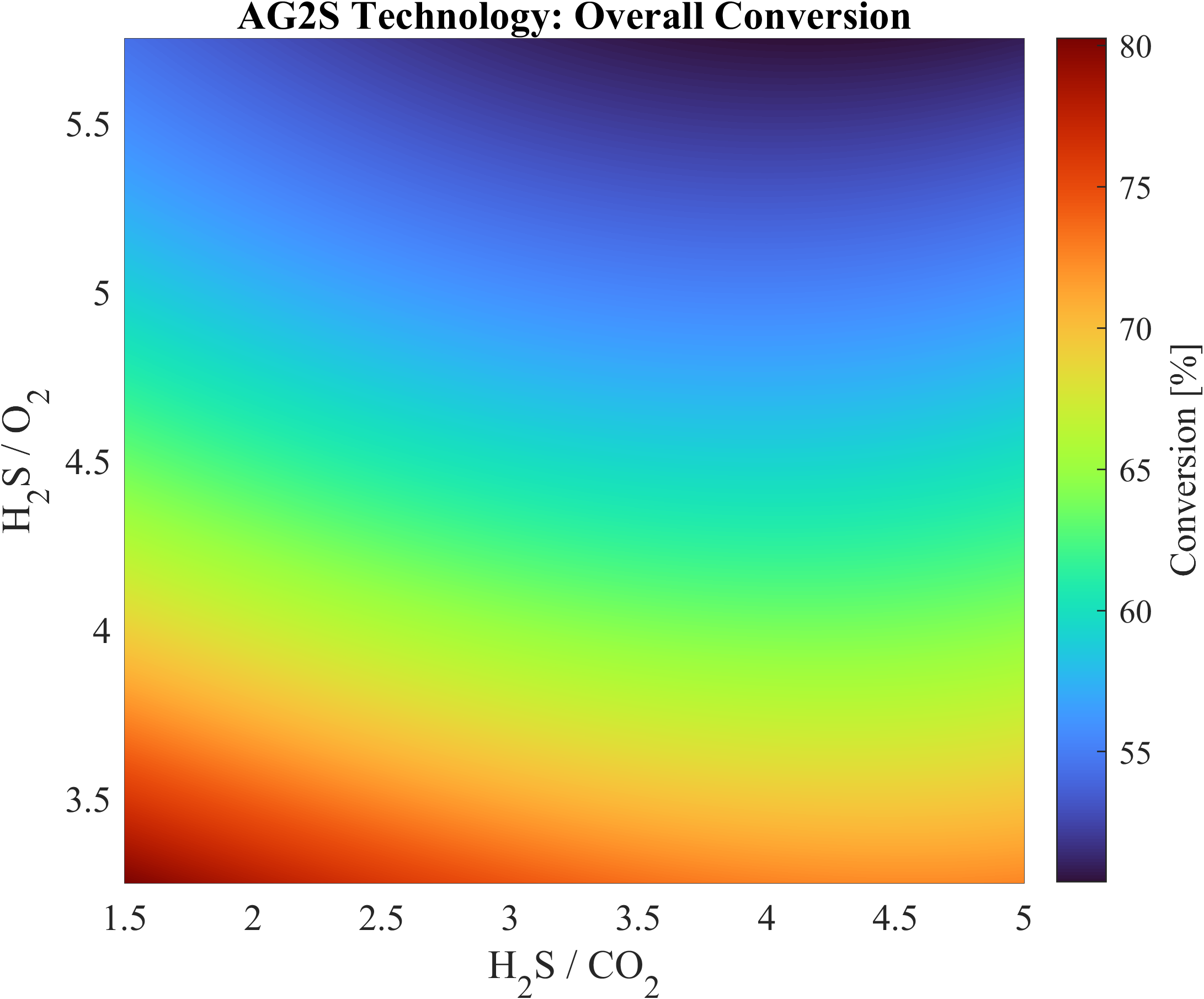

Supplement: Supplementary file 2 [file ie5c05101_si_002.zip › Images/plot_CONVERSION.png]

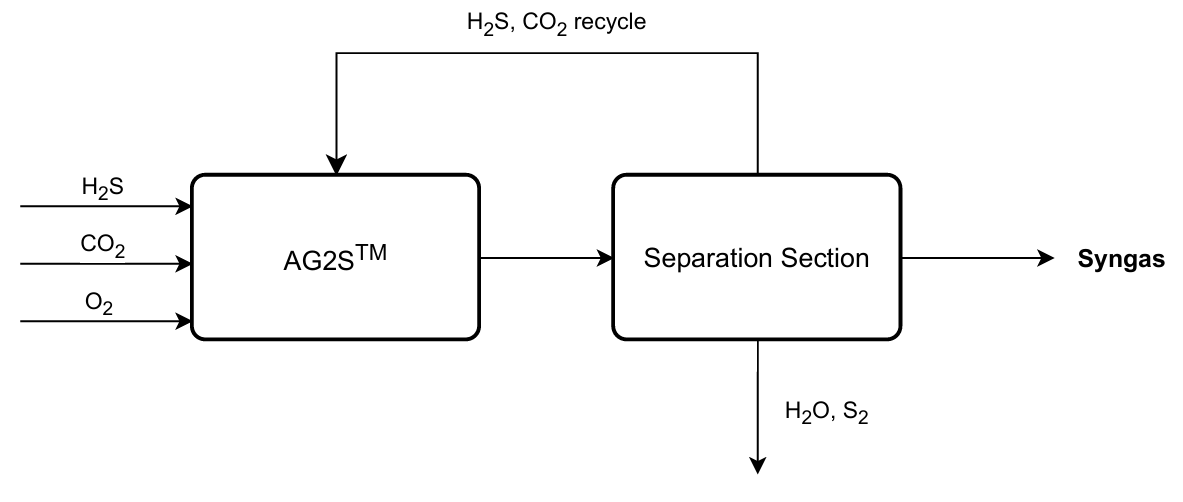

Supplement: Supplementary file 2 [file ie5c05101_si_002.zip › Images/0_BFD.png]

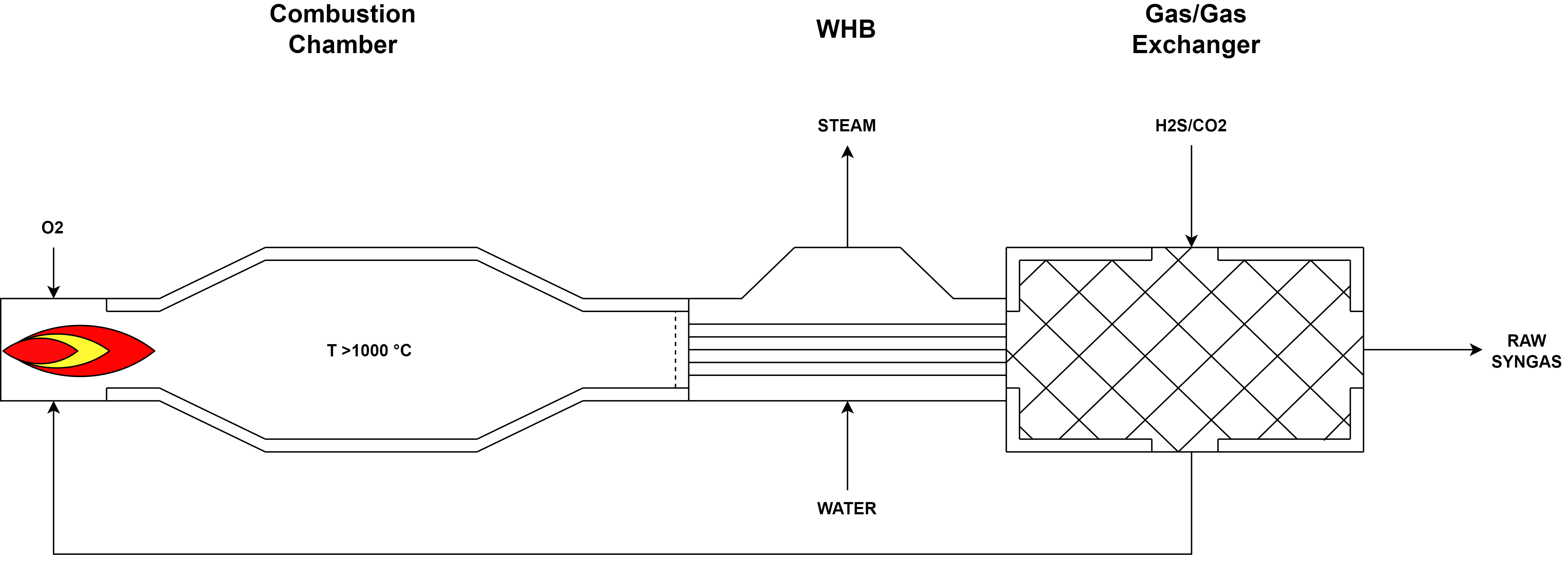

Supplement: Supplementary file 2 [file ie5c05101_si_002.zip › Images/2_RTR.png]

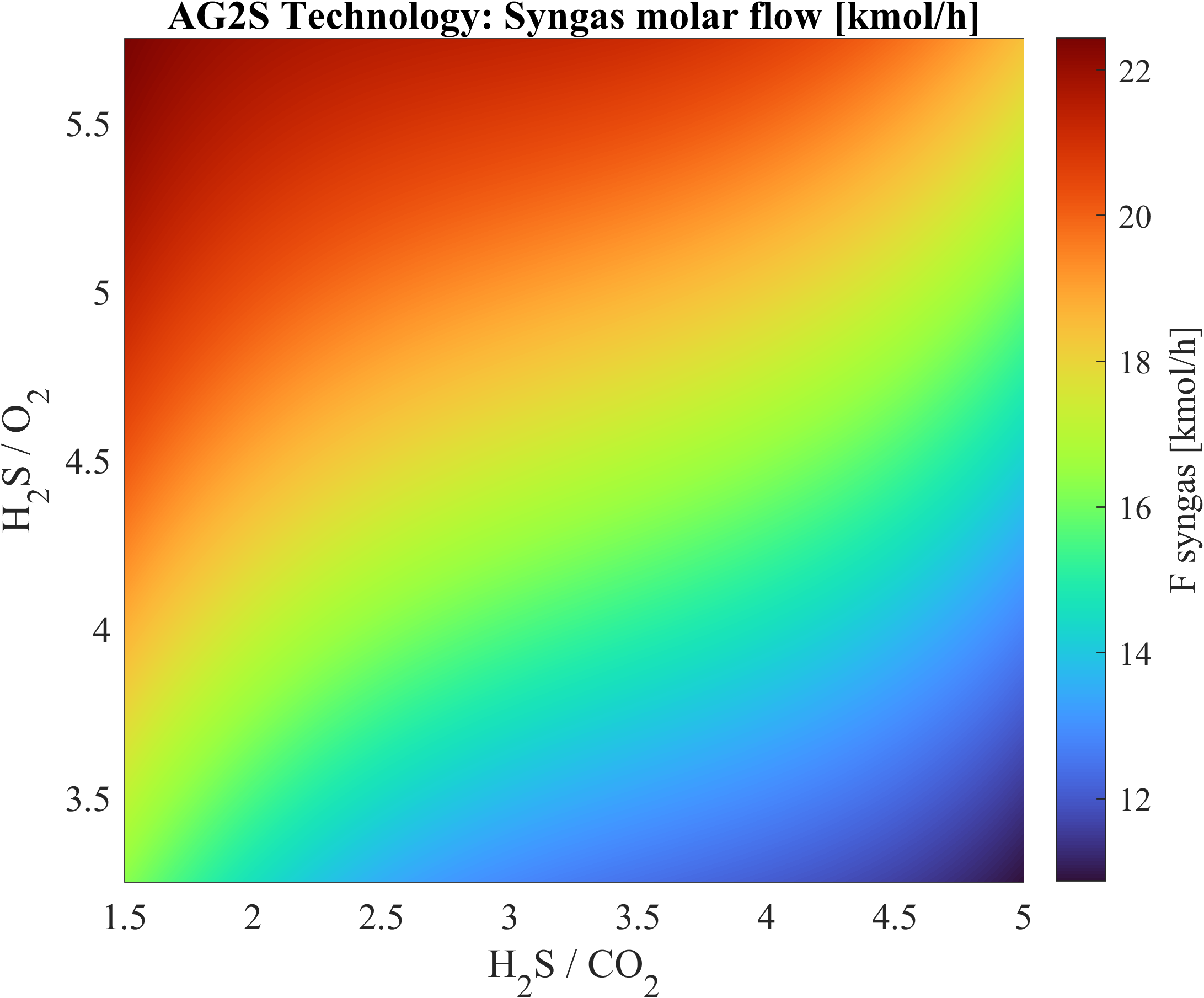

Supplement: Supplementary file 2 [file ie5c05101_si_002.zip › Images/PLOT_SYNGASmolarflow.png]

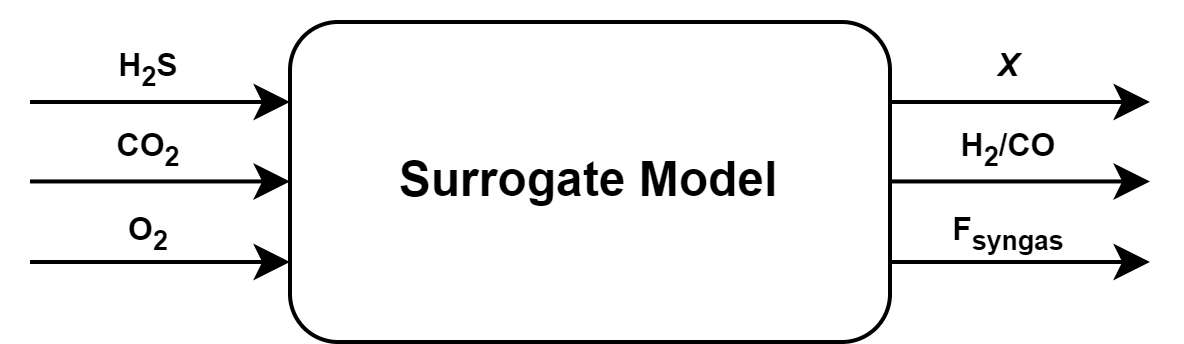

Supplement: Supplementary file 2 [file ie5c05101_si_002.zip › Images/SurrogateModel.png]

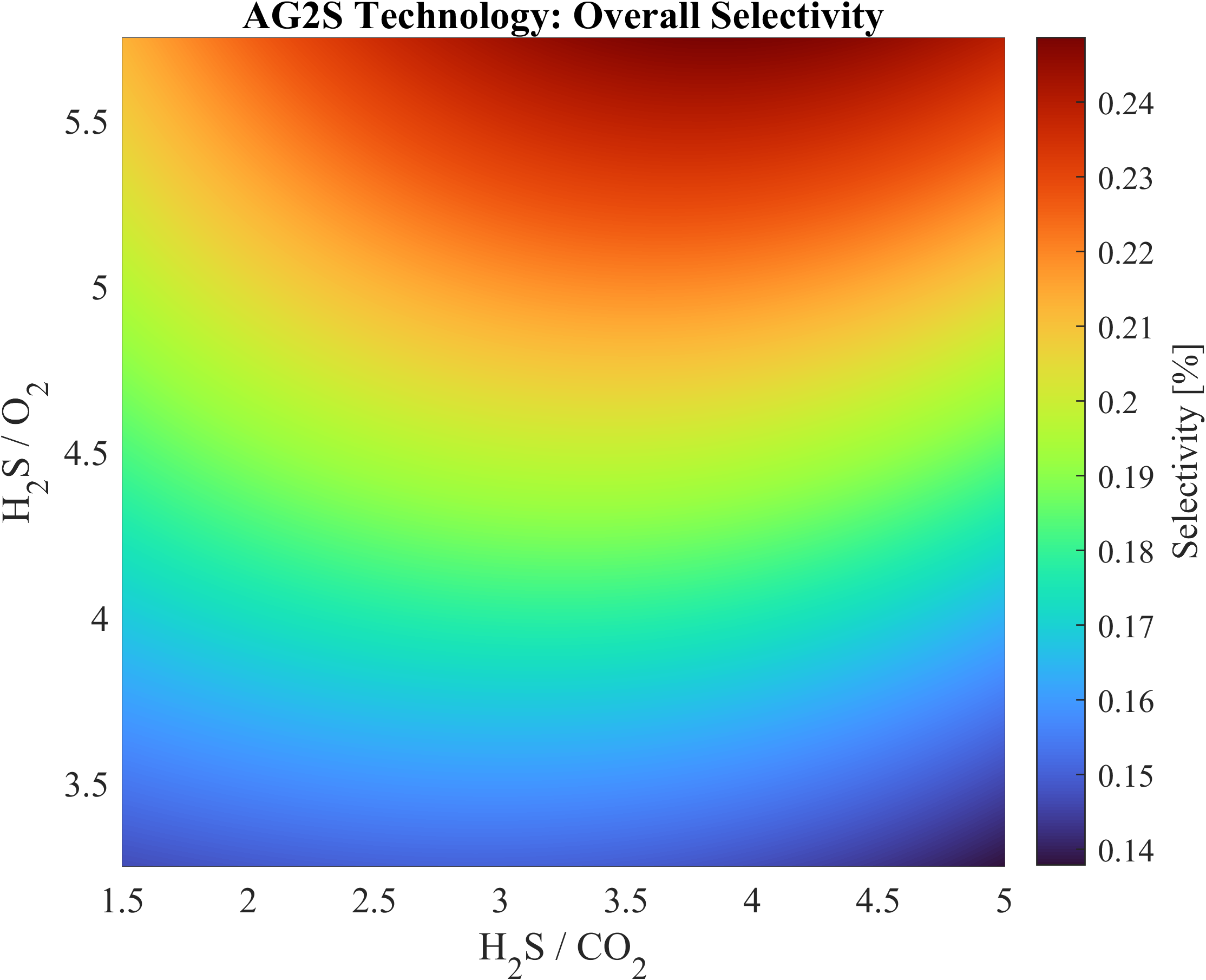

Supplement: Supplementary file 2 [file ie5c05101_si_002.zip › Images/plot_SELECTIVITY.png]

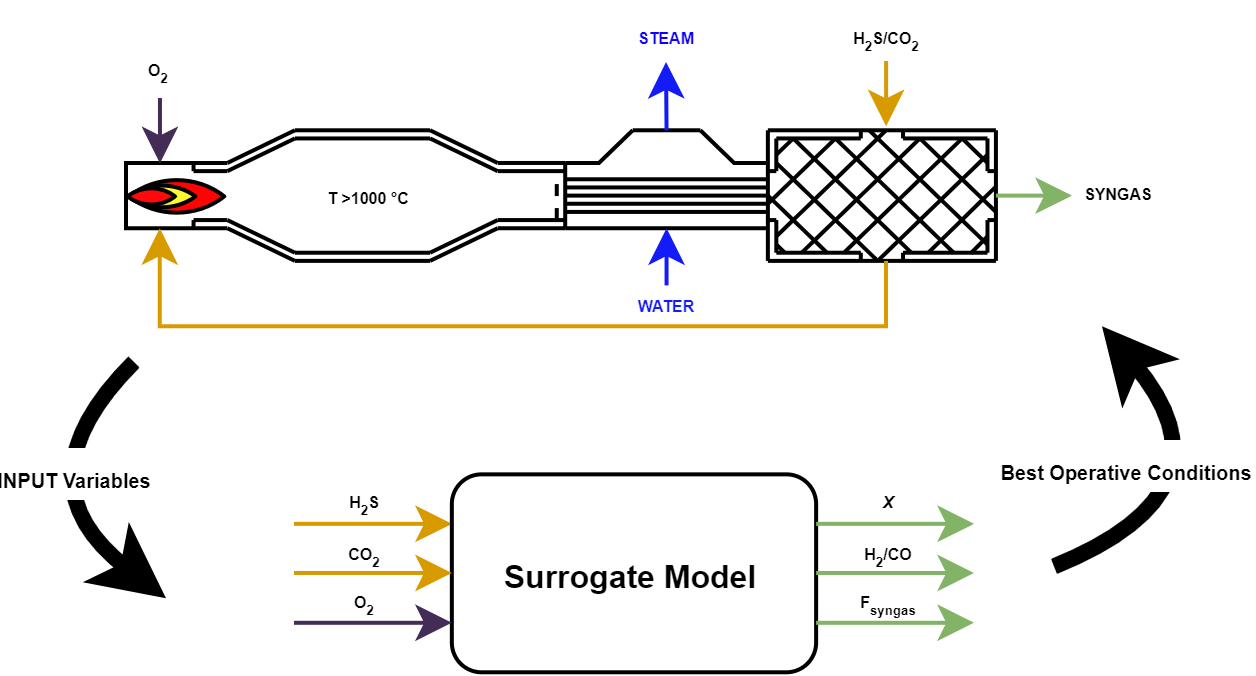

Supplement: Supplementary file 2 [file ie5c05101_si_002.zip › Images/TOC.png]

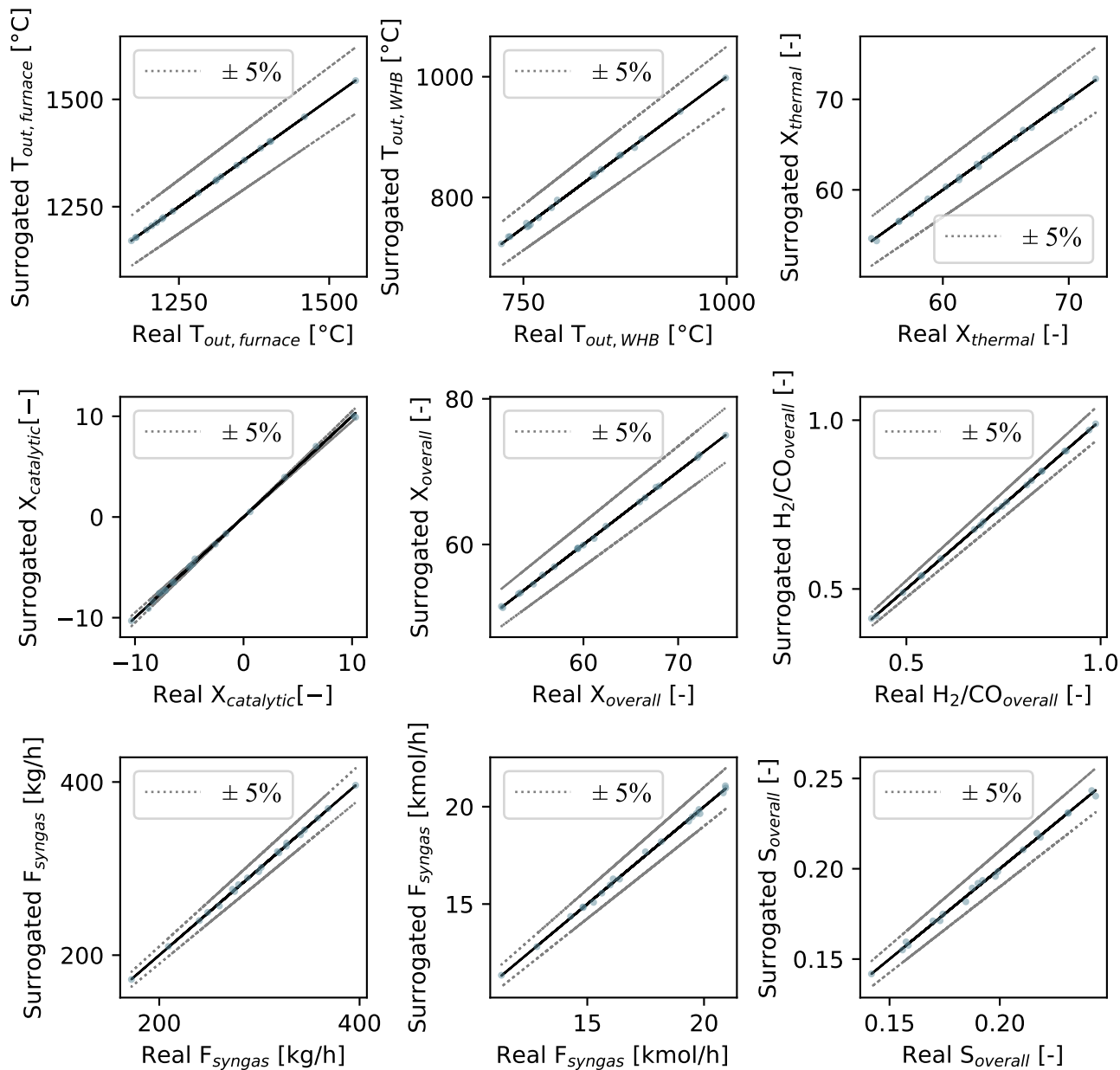

Supplement: Supplementary file 2 [file ie5c05101_si_002.zip › Images/6_realVSsurrogate.pdf]

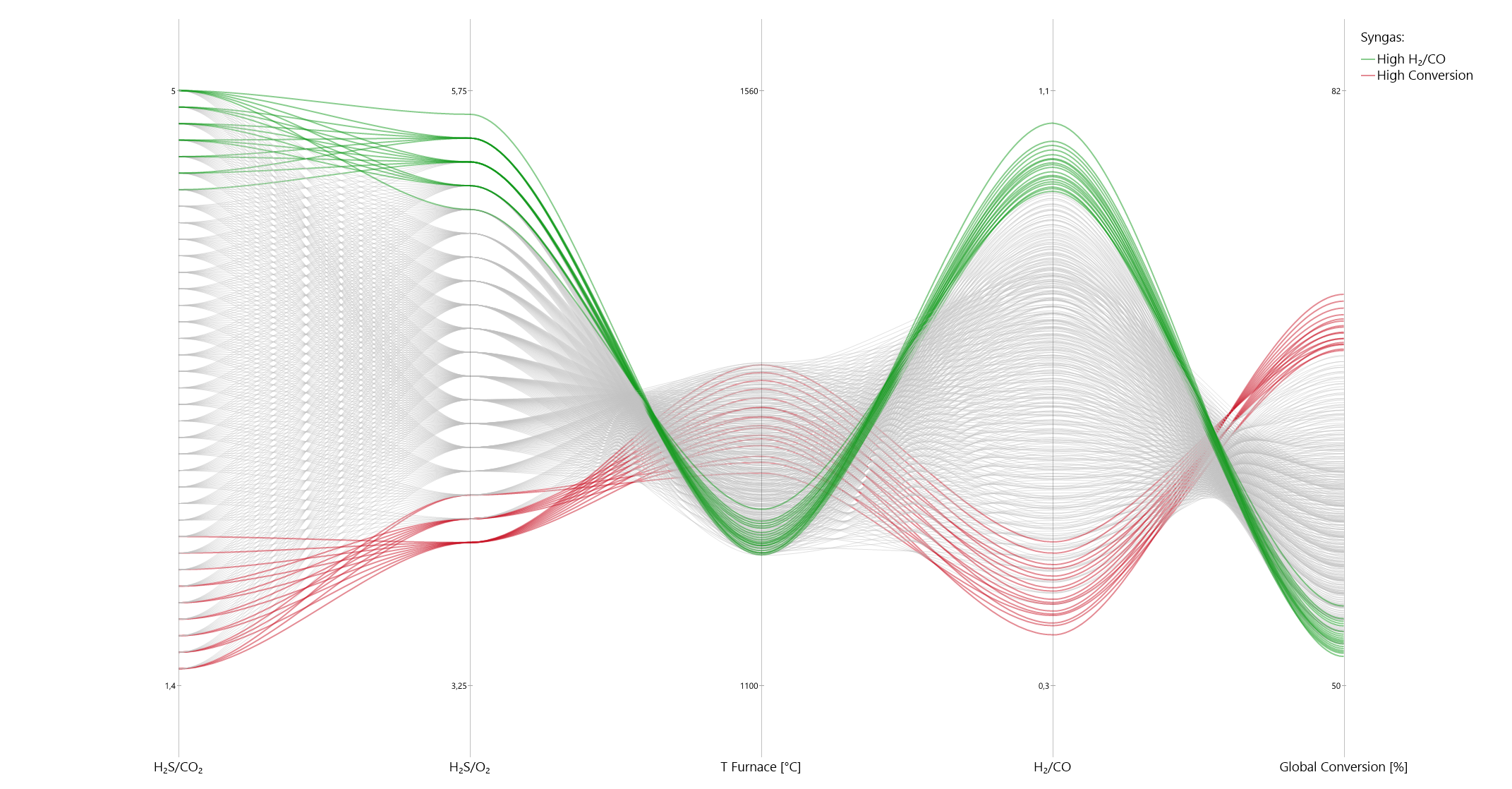

Supplement: Supplementary file 2 [file ie5c05101_si_002.zip › Images/ParallelPlot.png]

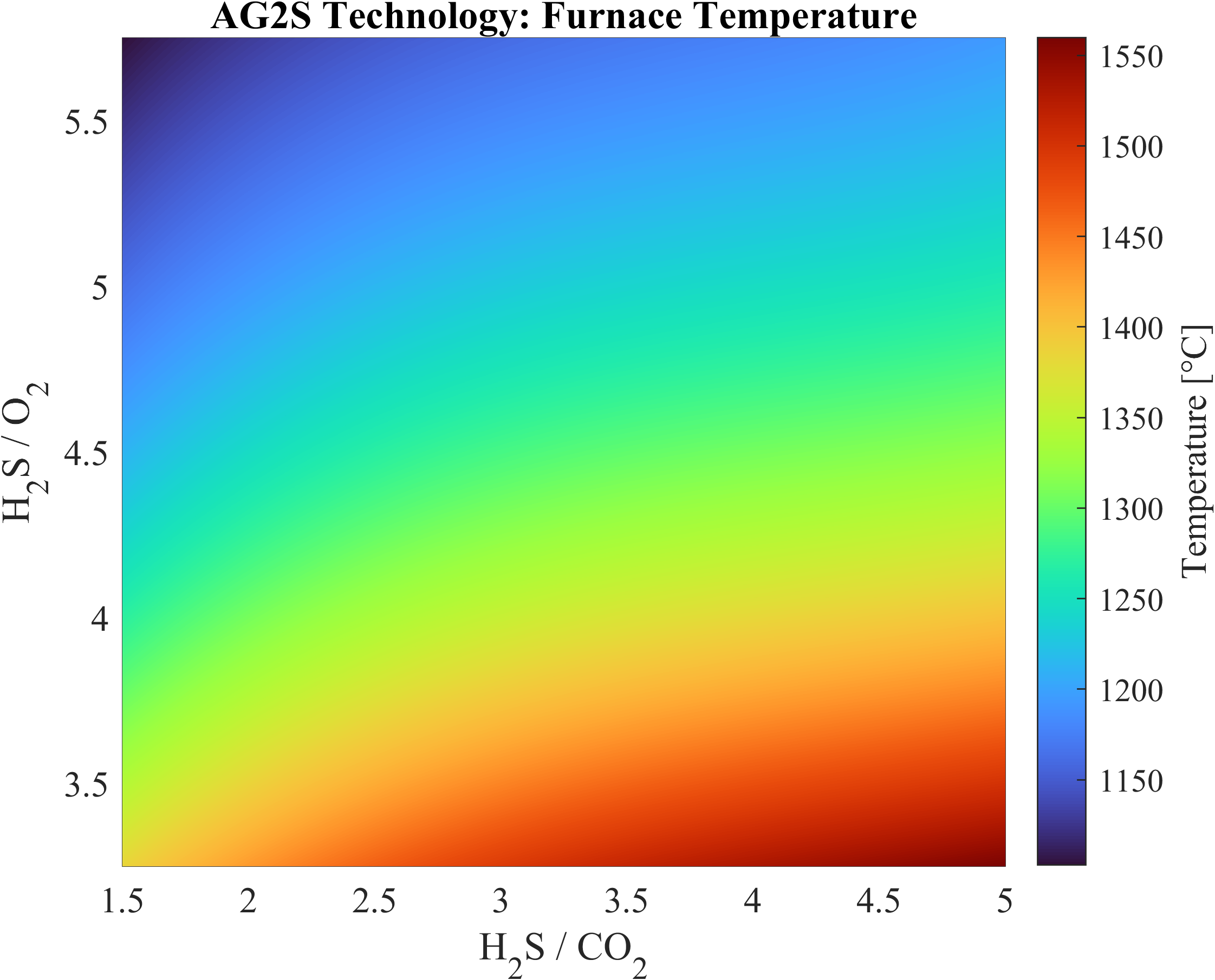

Supplement: Supplementary file 2 [file ie5c05101_si_002.zip › Images/plot_TEMPERATURE.png]

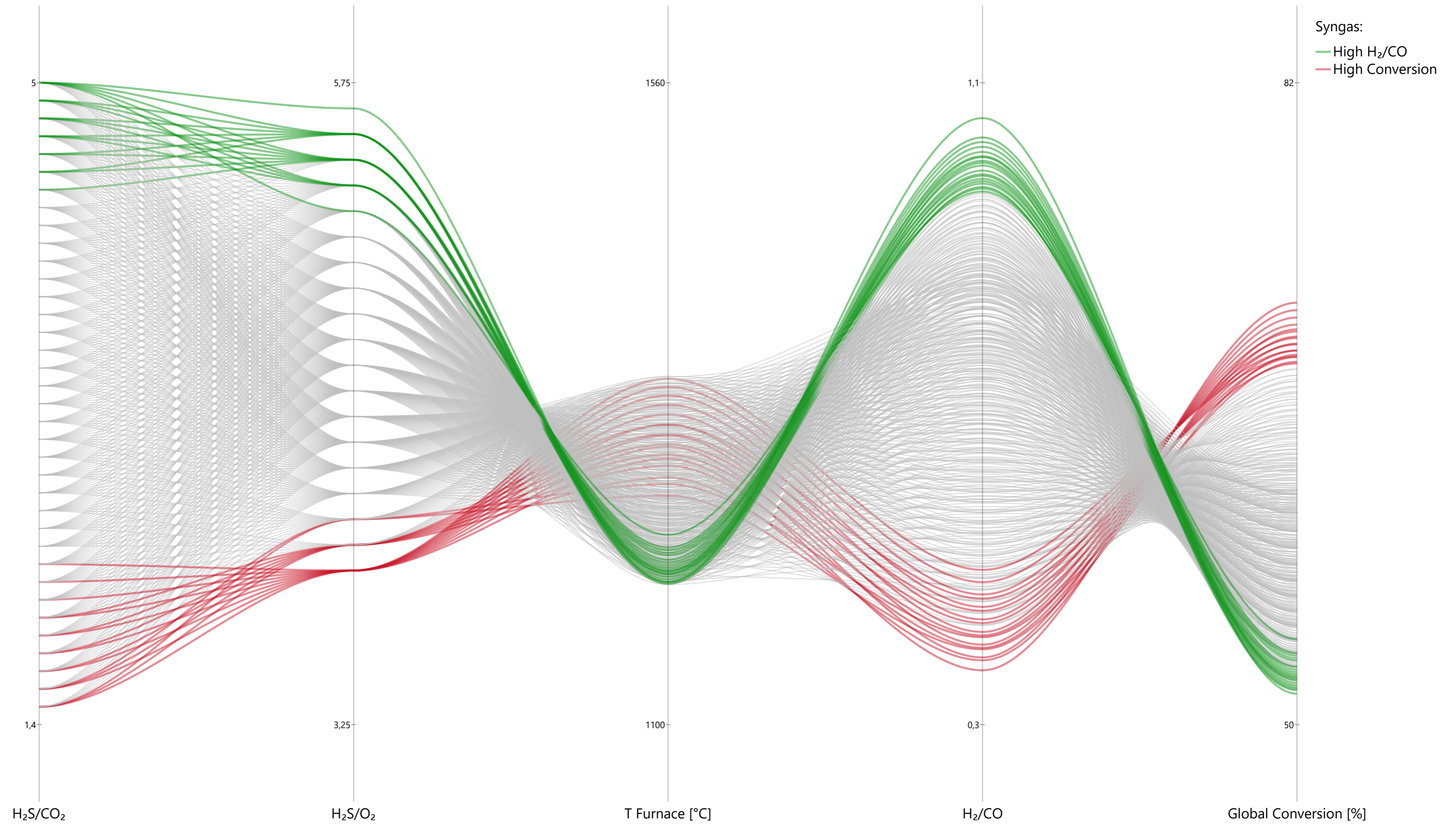

Supplement: Supplementary file 2 [file ie5c05101_si_002.zip › Images/ParallelPlot.pdf]

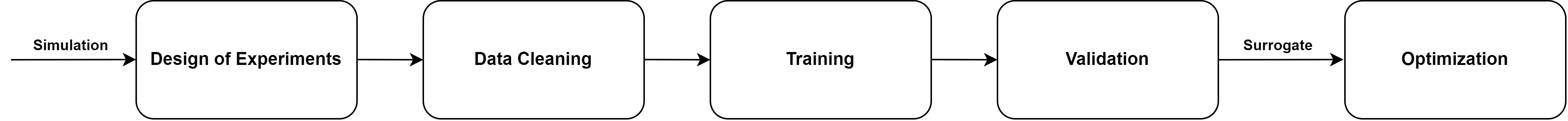

Supplement: Supplementary file 2 [file ie5c05101_si_002.zip › Images/WorkFlowSurrogate.png]
